# Supplementary material for: Archaeogenetic analysis of Neolithic sheep from Anatolia suggests a complex demographic history since domestication
Source: Commun Biol. 2021 Nov 12;4:1279. doi: 10.1038/s42003-021-02794-8 (PMC8589978; doi:10.1038/s42003-021-02794-8)
Supplement: Supplementary file 2 — Supplementary Information [file 42003_2021_2794_MOESM2_ESM.pdf]

Supplementary Information

Archaeogenetic analysis of Neolithic sheep from Anatolia suggests a complex demographic history since domestication

Erinç Yurtman[1+], Onur Özer[1,2+], Eren Yüncü[1+], Nihan Dilşad Dağtaş[1], Dilek Koptekin[3], Yasin Gökhan Çakan[4], Mustafa Özkan[1], Ali Akbaba[5], Damla Kaptan[1], Gözde Atağ[1], Kıvılcım Başak Vural[1], Can Yümni Gündem[6], Louise Martin[7], Gülşah Merve Kılınç[1,8], Ayshin Ghalichi[1,9], Sinan Can Açan[1], Reyhan Yaka[1], Ekin Sağlıcan[1], Vendela Kempe Lagerholm[10], Maja Krzewińska[10], Torsten Günther[11], Pedro Morell Miranda[11], Evangelia Pişkin[12], Müge Şevketoğlu[13], C. Can Bilgin[1], Çiğdem Atakuman[12], Yılmaz Selim Erdal[14,15], Elif Sürer[16], N. Ezgi Altınışık[14,15], Johannes A. Lenstra[17], Sevgi Yorulmaz[1], Mohammad Foad Abazari[18], Javad Hoseinzadeh[19], Douglas Baird[20], Erhan Bıçakçı[4], Özlem Çevik[21], Fokke Gerritsen[22], Rana Özbal[23], Anders Götherström[10#], Mehmet Somel[1#], İnci Togan[1#], Füsun Özer[14,15#]

[+] These authors contributed equally.
[#] These authors jointly supervised this work.

[1]Department of Biological Sciences, Middle East Technical University, Ankara, Turkey
[2]Emmy Noether Group Evolutionary Immunogenomics, Max Planck Institute for Evolutionary Biology, Plön, Germany
[3]Department of Health Informatics, Middle East Technical University, Ankara, Turkey
[4]Department of Prehistory, Istanbul University, Laleli, Istanbul, Turkey
[5]Department of Anthropology, Ankara University, Ankara, Turkey
[6]Department of Archaeology, Batman University, Batman, Turkey
[7]Institute of Archaeology, University College London, London, UK
[8]Department of Bioinformatics, Graduate School of Health Sciences, Hacettepe University, Ankara, Turkey
[9]Department of Archaeogenetics, Max-Planck Institute for the Science of Human History, Jena, Germany
[10]Archaeological Research Laboratory, Department of Archaeology and Classical Studies, University of Stockholm, Stockholm, Sweden
[11]Department of Organismal Biology, Human Evolution Research Program, Uppsala University, Uppsala, Sweden
[12]Department of Settlement Archaeology, Middle East Technical University, Ankara, Turkey
[13]Centre for Archaeology, Cultural Heritage and Conservation, Cyprus International University, Haspolat, Cyprus
[14]Department of Anthropology, Hacettepe University, Ankara, Turkey
[15]Molecular Anthropology Group (Human_G), Hacettepe University, Ankara, Turkey

[16]Department of Modeling and Simulation, Graduate School of Informatics, Middle East Technical University, Ankara, Turkey
[17]Faculty of Veterinary Medicine, Utrecht University, Utrecht, Netherlands
[18]Research Center for Clinical Virology, Tehran University of Medical Sciences, Tehran, Iran.
[19]Department of Archaeology, University of Kashan, Kashan, Iran
[20]Department of Archaeology, Classics, and Egyptology, University of Liverpool, Liverpool, UK
[21]Department of Archaeology, Trakya University, Edirne, Turkey
[22]Netherlands Institute in Turkey, Istanbul, Turkey
[23]Department of Archaeology and History of Art, Koç University, Istanbul, Turkey

Supplementary Tables

Supplementary Table 1: Modern sheep breeds and ancient sheep obtained from literature and used in the genomic analyses of the present study. Names, abbreviations and geographic origins are from Kijas et al.[1] and Taylor et al.[2]

Abbreviation	Modern Breeds	Origin	
CHA	Changthangi	S Asia	Non-European
IDC	Deccani	S Asia	
AFS	Afshari	SW Asia	
NDZ	Norduz	SW Asia	
CFT	Cyprus Fat Tail	East Mediterranean Island	
CHI	Chios	East Mediterranean Island	
SKZ	Sakız	East Mediterranean Island	
EMZ	Ethiopian Menz	Africa	
COM	Comisana	SE Europe	European
LEC	Leccese	SE Europe	
BOS	Bundner Oberlander Sheep	Central Europe	
ERS	Engadine Red Sheep	Central Europe	
VBS	Valais Blacknose Sheep	Central Europe	
FIN	Finn sheep	Northern Europe	
NSO/NSP	Old Norwegian Spælsau	Northern Europe	
CHU	Churra	SW Europe	
MER	Australian Merino	SW Europe	
SAB	Sardinian Ancestral Black	SW Europe	
**Abbreviation**	**Ancient Sheep**	**Origin**	
OBI	Obishir V	Southern Kyrgyzstan	

Supplementary Table 2: The number of SNPs in each length class for each ancient sample after PMDtools[3] filtering.

Sample ID	No Filter	Only Transversion	Less than 70 bp	More than 100 bp	PMD filtered
TEP03	15794	5492	3080	14716	11207
TEP62	20281	5843	5347	17983	13823
TEP83	6212	2349	1032	3767	3482
ULU31	8533	3437	6716	988	5172

Supplementary Table 3: Phenotype-related SNPs in ancient individuals. Reference allele frequencies across n=749 modern domestic, n=6 Argali and n=4 Vignei sheep, as well as genotypes of four Anatolian Neolithic sheep individuals at 18 SNP markers linked to putatively positively selected loci in domestic sheep[1]. The "Ref/Alt" column indicates reference and alternative alleles. The alleles assigned as alternatives are shown in bold. Argali (*Ovis ammon*) and Vignei (*Ovis vignei*) sheep were included for ancestral state inference (e.g., at SNP position OAR6_76473607.1, both Argali and Vignei carry the alternative allele C with a frequency of 1, so the ancestral state may be inferred to be C). Ancestral states could be inferred in 16 loci. Empty cells indicate that the allele has not been detected in an ancient individual. The modern genotype frequencies are calculated based on data from Kijas et al.[1] Genes in putatively selected regions and their functional annotations are shown in parentheses (adopted from Kijas et al.[1]).

Chr	SNP ID	Ref / Alt	Modern domestic (*n*=749)	Argali (*n*=6)	Vignei (*n*=4)	TEP03	TEP62	TEP83	ULU31
2	s29378.1	A/**G**	0.96	1.00	1.00	-	A	A	A
2	s20468.1 (*NPR2*: skeletal morph,body size)	G/**A**	0.51	1.00	1.00	A	A	-	A
2	s01865.1	T/**C**	0.96	1.00	1.00	T	T	-	-
3	OAR3_141586525.1	C/**T**	0.67	0.92	0.50	C	C	-	C
5	s36709.1	A/**G**	0.97	1.00	1.00	A	A	-	-
6	s21552.1 (*FGF5*: hair variation in dogs)	G/**A**	0.87	1.00	1.00	-	G	-	G
6	OAR6_40277406.1	G/**A**	0.96	1.00	1.00	G	G	-	G
6	OAR6_76473607.1 (*KIT*: pigmentation in cattle, pigs)	**T**/C	0.59	0.00	0.00	C	T	-	C
7	s69881.1	A/**C**	0.998	1.00	1.00	A	A	A	A
8	OAR8_67529714.1	**G**/T	0.52	0.00	0.00	T	T	G	-
10	OAR10_29511510.1 (*RXFP2*: horn absence)	C/T	0.89	0.92	0.25	C	T	-	T
11	OAR11_18701428.1	**A**/G	0.72	0.00	0.00	G	G	-	A
13	OAR13_51852034.1 (*BMP2*: skeletal morph,body size)	G/**A**	0.90	1.00	1.00	G	A	-	-
16	OAR16_41943180.1 (*PRLR*: milk traits in cattle)	A/**C**	0.63	1.00	1.00	A	A	A	-
17	s41543.1	C/**T**	0.98	1.00	1.00	C	C	-	-
19	s38567.1	**A**/G	0.76	0.00	0.00	G	G	G	-
25	s03686.1	**T**/C	0.45	0.00	0.00	T	C	-	-
25	s10489.1	A/G	0.87	0.17	0.62	-	A	A	A

Supplementary Table 4: Mitochondrial haplogroup (HPG) frequencies of three present-day sheep breeds from Anatolia and different regions of Eurasia. References are shown in the "Source" column. The frequency of the most common haplogroup in each sample is shown in bold.

Region<br>(breed) (sample size)	HPG A<br>(%)	HPG B<br>(%)	HPG C<br>(%)	HPG D<br>(%)	HPG E<br>(%)	Source
China<br>(*n*=463)	**56**	35	9	0	0	Lv et al.[4]
Mongolian Plateau<br>(*n*=245)	**56**	28	16	0	0	Luo et al.[5]<br>Lv et al.[4]
Indian Subcontinent<br>(*n*=500)	**77**	20	3	0	0	Singh et al.[6]<br>Gorkhali et al.[7]<br>Lv et al.[4]
Central Anatolia<br>(Akkaraman) (*n*=50)	26	**52**	14	2	6	Demirci et al.[8]
West Anatolia<br>(Sakız) (*n*=49)	4	**88**	8	0	0	Demirci et al.[8]
Northwest Anatolia<br>(Kıvırcık) (*n*=45)	0	**96**	4	0	0	Demirci et al.[8]
East Europe<br>(*n*=126)	8	**92**	0	0	0	Tapio et al.[9]<br>Ćinkulov et al.[10]<br>Lv et al.[4]
Central Europe<br>(*n*=19)	21	**79**	0	0	0	Tapio et al.[9]
West Europe<br>(*n*=177)	4	**94**	2	0	0	Pereira et al.[11]<br>Tapio et al.[9]

Supplementary Table 5: Mitochondrial DNA control region marker variants. mtDNA nucleotide positions used for haplogroup assignment are based on coordinates of the *Ovis aries* mitogenome AF010406 (NCBI GenBank). Table adapted from Demirci et al.[8]

Haplogroup	Reference sequence	Positions and identities of bases used for haplogroup assignment				
		15459	15476	15484	15509	15512
HPG A	HM236174	T	T	A	A	T
HPG B	HM236176	C	T	G	A	T
HPG C	HM236178	C	T	G	G	T
HPG D	HM236180	C	T	G	A	C
HPG E	HM236182	C	C	G	G	T

Supplementary Table 6: Mitochondrial DNA amplification and sequencing success rates. Success refers to aDNA fragments being amplified and sequenced at least twice, independently.

Archaeological site	Number of samples studied	Number of successful samples (with at least 2 amplifications)	Success rate
Ulucak Höyük	40	9	22.5%
Barcın Höyük	44	17	39%
Canhasan III	5	3	60%
Tepecik-Çiftlik Höyük	69	41	59%
Boncuklu Höyük	5	1	20%
Pınarbaşı	17	8	47%
Total	180	79	44%

Supplementary Table 7: Mitochondrial haplogroup (HPG) frequencies of seven ancient sheep breeds from Anatolia, based on samples with at least two sequences (present study) and Kyrgyzstan (Obishir V)[2]. The frequency of the most common haplogroup in each sample is shown in bold.

Archaeological site	HPG A (%)	HPG B (%)	HPG C (%)	HPG D (%)	HPG E (%)
Boncuklu Höyük (n=1)	0	**100**	0	0	0
Pınarbaşı (n =8)	0	**87**	0	13	0
Tepecik_Çiftlik (n = 8)	7	**78**	0	5	10
Canhasan (n = 3)	0	**100**	0	0	0
Barcın (n = 17)	6	**94**	0	0	0
Ulucak (n= 9)	0	**78**	0	11	11
Kyrgyzstan (Obishir V) (*n*=3)	**100**	0	0	0	0

Supplementary Table 8: Comparison of haplogroup distributions within Anatolian ancient samples (Supplementary Table 7) and modern samples from Anatolia, Asia and Europe (Supplementary Table 4) using Fisher's exact test. The p-values were adjusted for multiple testing using the BH correction based on the total number of comparisons (n=17). Data from Sakız and Kıvırcık are pooled as modern-day west Anatolia. "Ancient" samples include all individuals with at least two amplifications (Supplementary Data 1). Tepecik Çiftlik, Boncuklu, Pınarbaşı and Canhasan III Höyük samples are pooled as ancient central Anatolian samples, Barcın and Ulucak Höyük samples are pooled as ancient western Anatolian samples. The effect size of frequency differences is measured by Cohen's *w*[12], which is traditionally considered "small" when $w < 0.10$ and "large" when $w > 0.50$.

**Regions compared**	**Central Anatolia (ancient) (n=53)**		**West Anatolia (ancient) (n=26)**	
	w	***p*-value**	***w***	***p*-value**
China (n=436)	0.47	<0.001	0.38	<0.001
Mongolia (n=245)	0.57	<0.001	0.48	<0.001
India (n=500)	0.56	<0.001	0.45	<0.001
East Europe (n=126)	0.31	0.001	0.26	0.052
Central Europe (n=19)	0.29	0.155	0.32	0.155
West Europe (n=177)	0.33	<0.001	0.26	0.055
West Anatolia (modern) (n=94)	0.34	0.001	0.28	0.073
Central Anatolia (modern) (n=50)	0.42	<0.001	0.40	0.008
Central Anatolia (ancient) (n=53)	-	-	0.10	0.944

Supplementary Figures

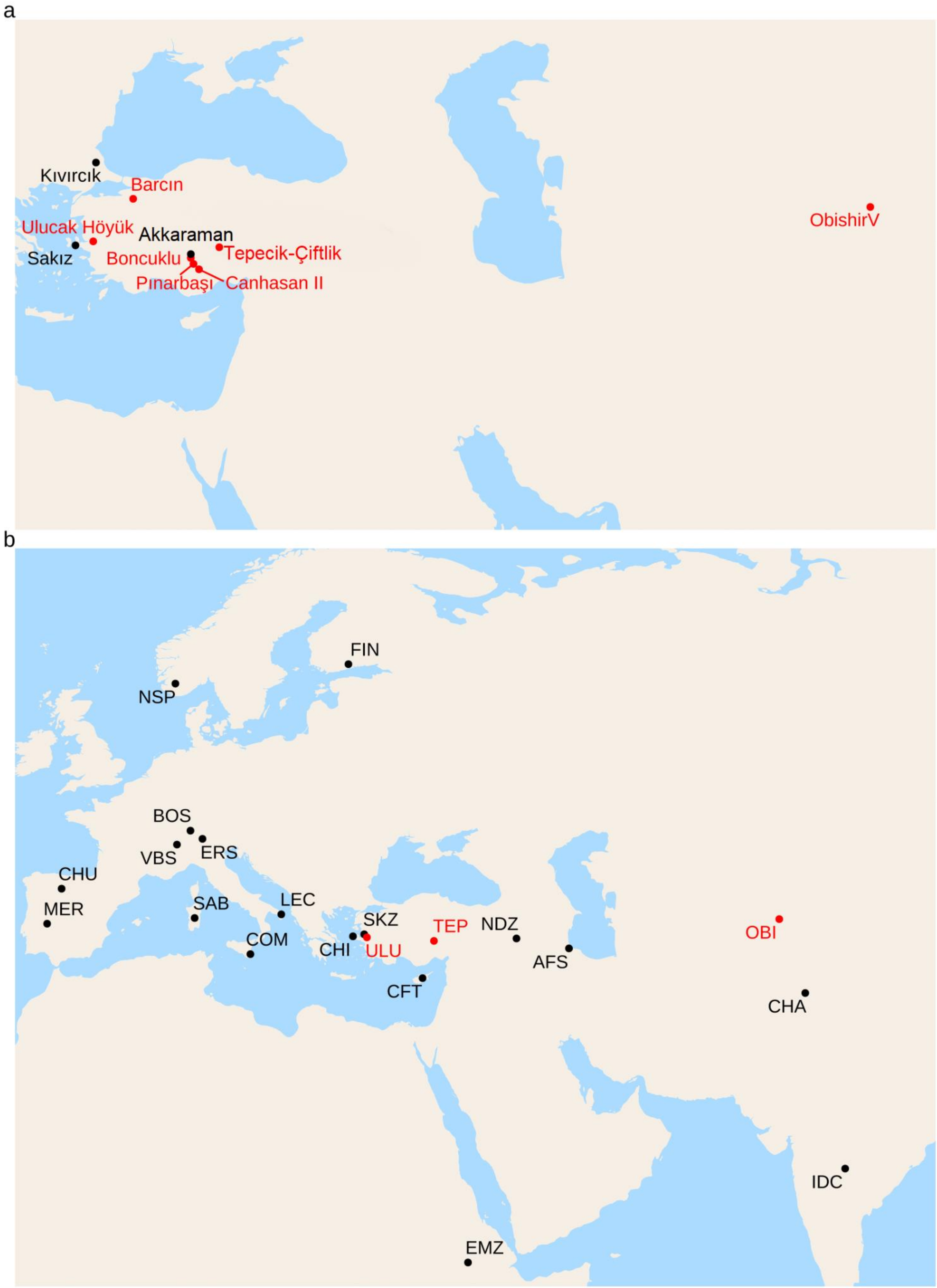

Supplementary Fig. 1: Geographic map of modern and ancient sheep samples in this study. a) Geographic locations of modern breeds (in black), ancient Anatolian sheep individuals (red) and ancient Kyrgyzstan, Obishir sheep individuals (red) used in mitochondrial DNA analyses. b) Geographic locations of modern breeds (black) and ancient individuals (red) used in genomic analyses. For population abbreviations see Supplementary Table 1.

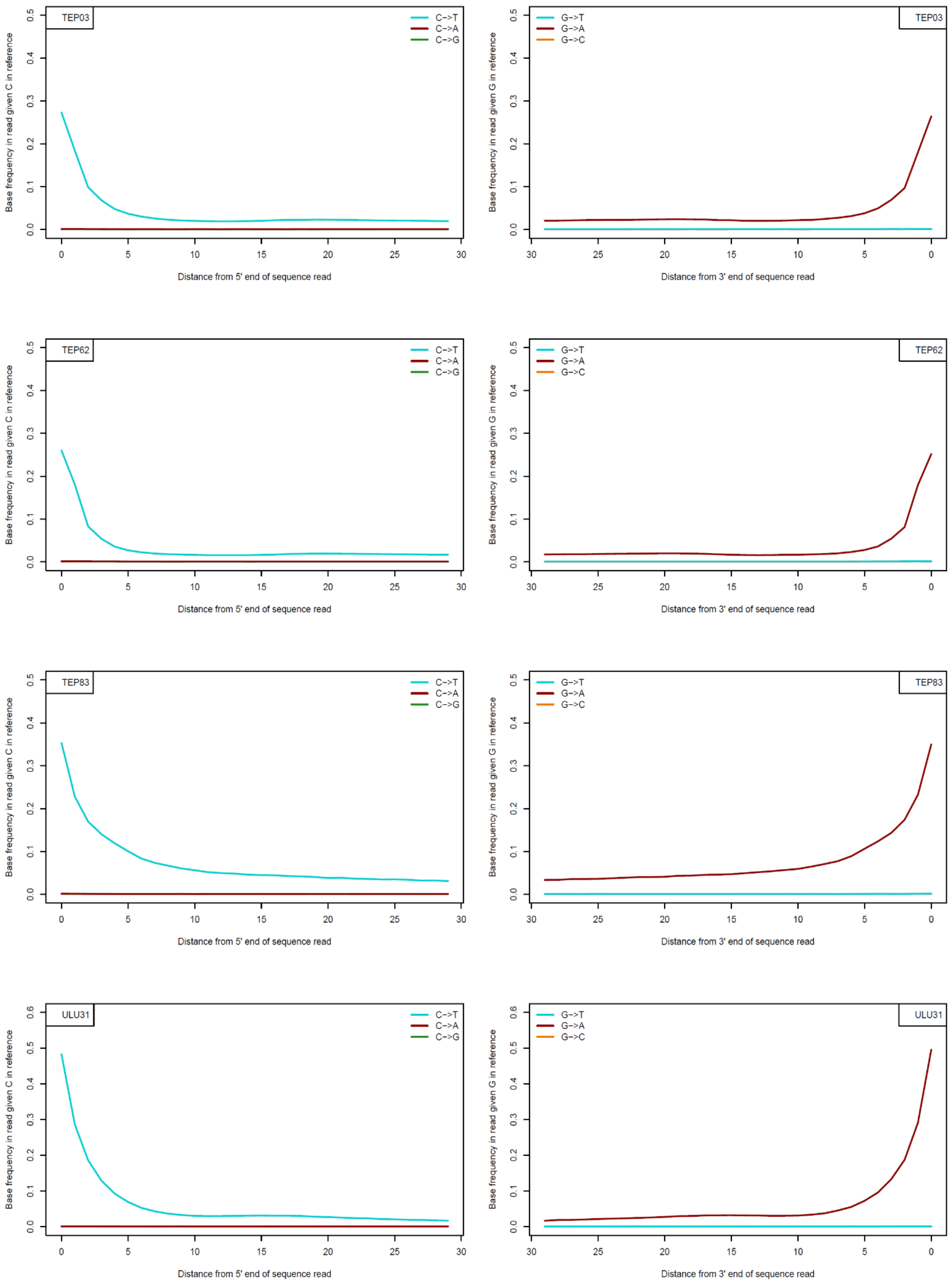

Supplementary Fig. 2: Nucleotide mismatch patterns in Anatolian Neolithic sheep libraries used in genomic analyses. Postmortem damage for TEP03, TEP62, TEP83, and ULU31, from top to bottom. The y-axes indicate the frequency of mismatches between sequenced reads and the reference genome as a function of the distance (x-axis) from 5' ends (left panels) or 3' ends (right panels) of the reads for each of the Anatolian Neolithic sheep individual used in population genomic analyses. The mismatch types are indicated in the inset.

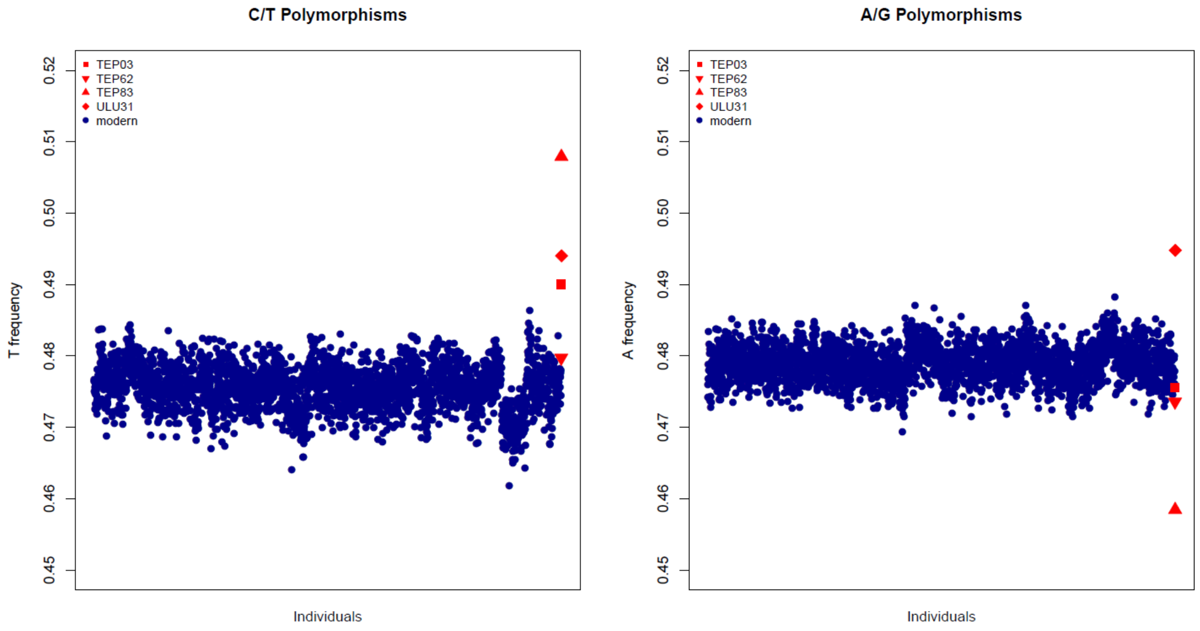

Supplementary Fig. 3: Frequency of damaged alleles at transition sites in Anatolian Neolithic sheep vs. modern-day breed genotypes. For each ancient and modern individual, proportion of A at A/G polymorphisms, and proportion of T at C/T polymorphisms are shown. Blue points indicate the genotypes of modern-day breeds (n=749), while ANS genotypes (obtained after trimming) are shown in red.

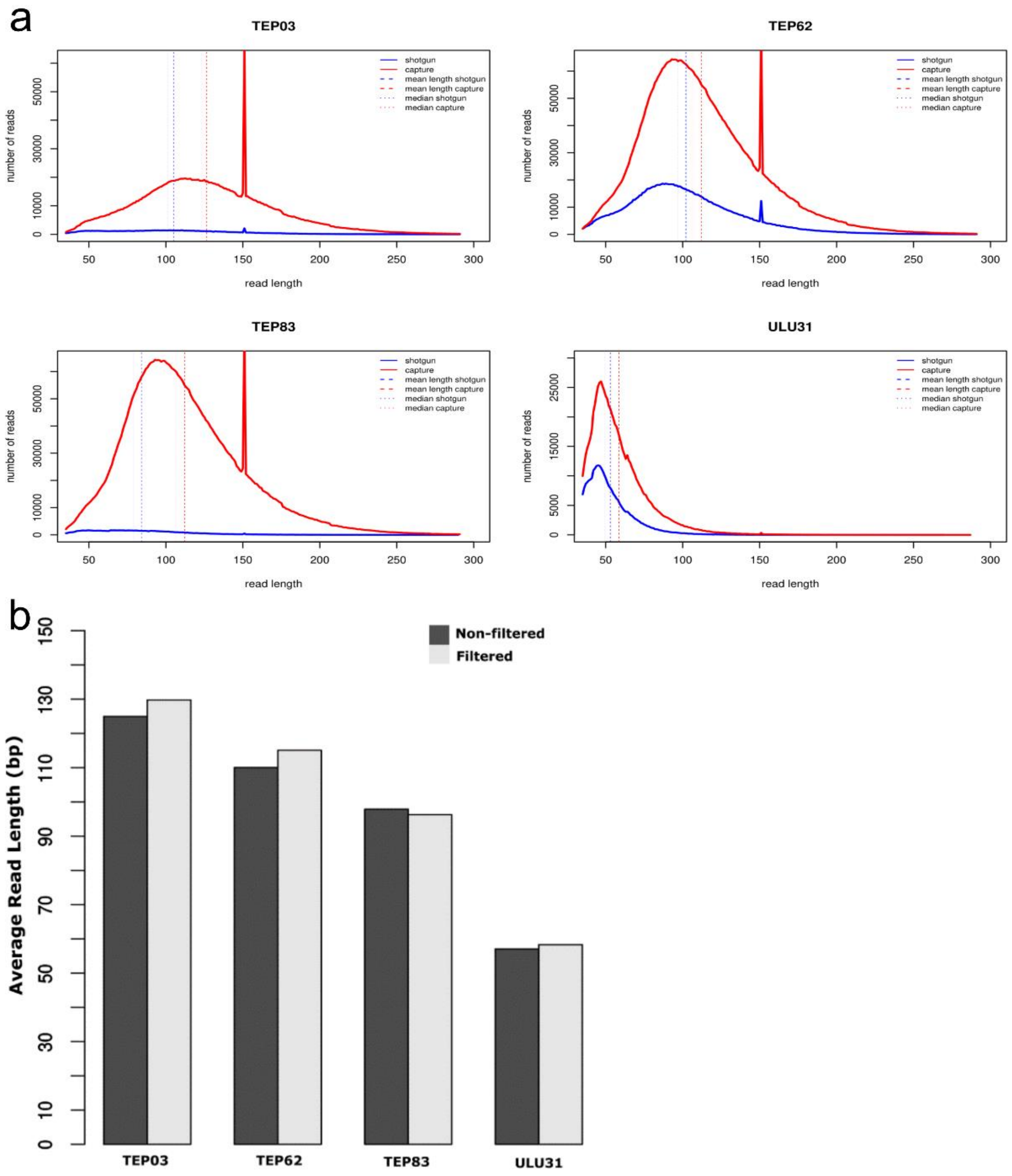

Supplementary Fig. 4: Average read lengths and frequency distributions of read lengths of Anatolian Neolithic sheep libraries. a) The graphs represent lengths of reads that are mapped to the sheep genome obtained from direct shotgun sequencing (blue) and sequencing after hybridization capture (red) of libraries of ANS individuals. The spike at 150 bp is caused by the rare cases of fragments > 300 bp long; in this case, the forward and backward reads from these fragments do not merge (as sequencing is run for 150 cycles) and are amassed at 150 bp, creating the spike. b) Average read lengths before and after filtering for postmortem damage are calculated for all reads in ANS libraries (dark grey) or only 27-53% (median 41%) of reads that pass postmortem damage (based on the C to T signature) threshold 3 set by PMDtools[3] (light grey). We observe no trend of shorter molecule length among filtered reads that bear postmortem-induced damage, which would be expected if long molecules represented modern DNA contamination.

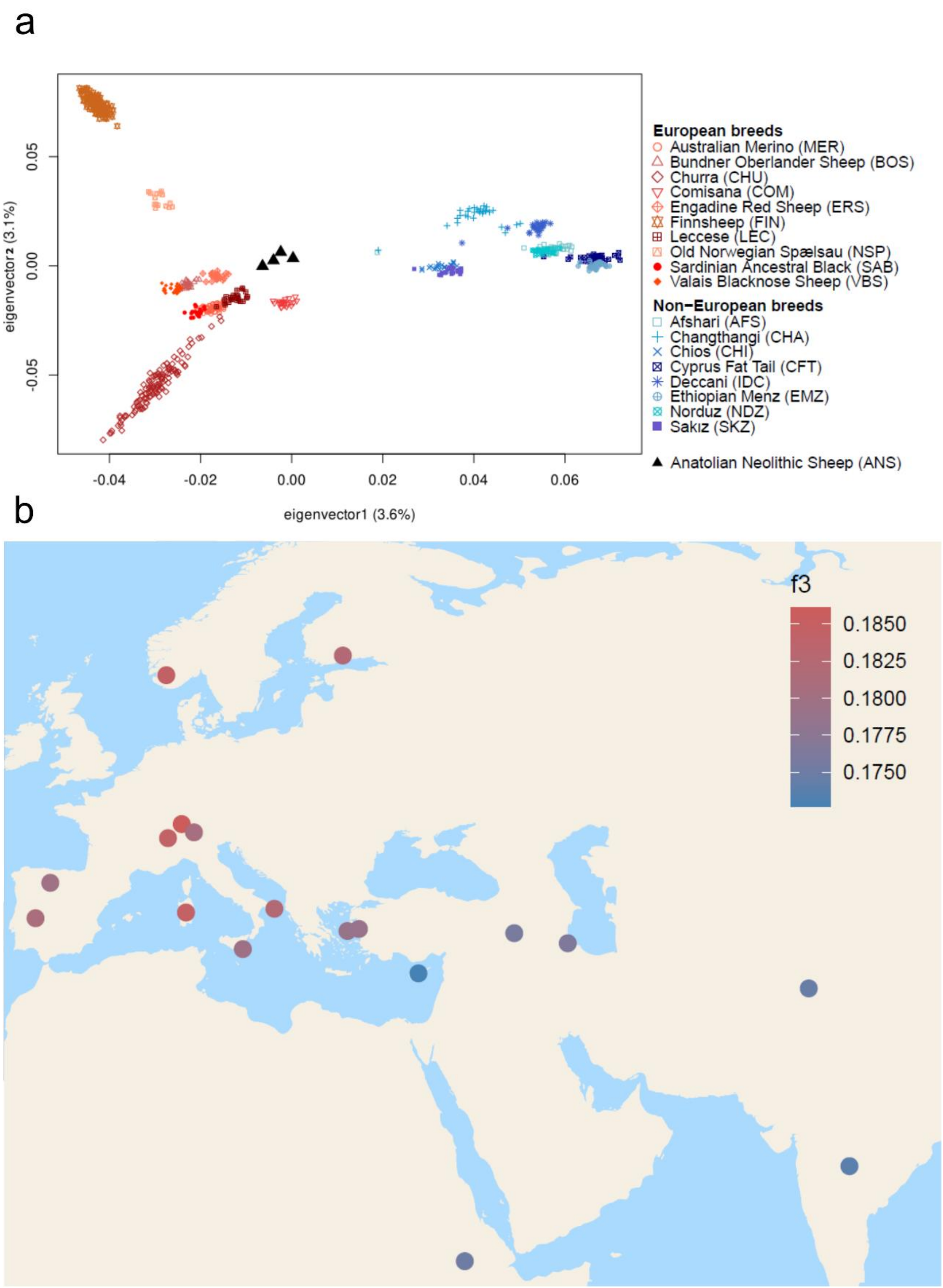

Supplementary Fig. 5: Principal components analysis and outgroup f_3 plots of Anatolian Neolithic sheep and modern sheep breeds using reads after filtering for postmortem damage. The panels a) and b) were produced in the same way as Figures 3 and 4, respectively, but using only the 27-53% (median 41%) that pass postmortem damage threshold 3 set by PMDtools[3].

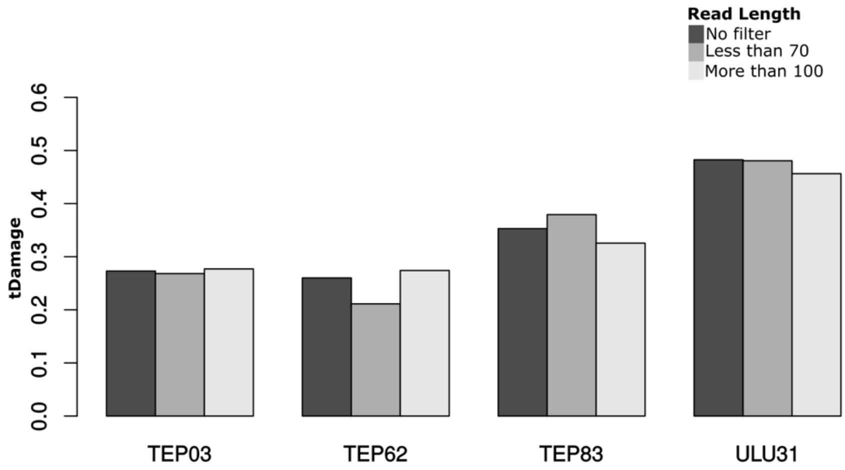

Supplementary Fig. 6: C to T mismatch values at the 5' ends calculated for all, short and long molecules of ANS libraries. The barplot shows postmortem-induced C to T mismatch proportions at 5' ends across all reads ("No filter") (black), short reads <70 bp (dark grey) and long reads >100 bp (light grey), of each Anatolian Neolithic sheep (ANS) library. We did not observe lower C to T mismatch proportions at 5' ends for long (>100 bp) reads.

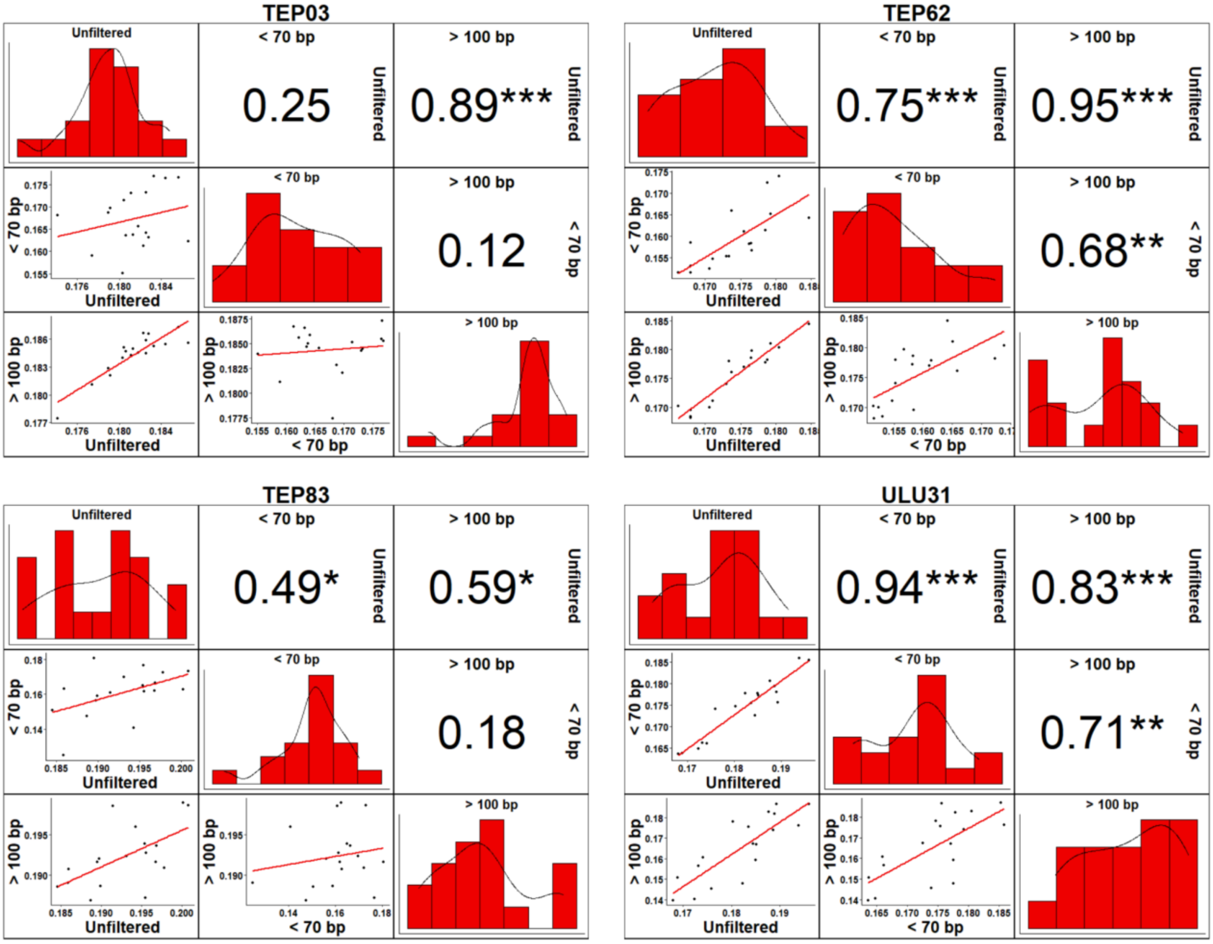

Supplementary Fig. 7: Comparison of outgroup f_3-statistics calculated for all (unfiltered), short (< 70 bp) and long (> 100 bp) molecules of each ANS individuals. We calculated f_3*(Argali; ANS*$_i$*, Modern)* using different molecule sets in the merged (shotgun and capture joined) libraries of each ANS individual (TEP03, TEP62, TEP83 and ULU31). In the scatter plots presented in the lower triangle of each panel, each point represents an f_3 value calculated as f_3*(Argali; ANS*$_i$*, Modern)* where *Modern* is a modern breed, and the two axes represent f_3 values calculated from different molecule sets (all, short, or long). The red line represents linear regression. Panels in the upper triangle of each panel show the Spearman correlation coefficients for the corresponding scatter plots; asterisks indicate significance of the Spearman test ('***': $p<0.001$, '**': $p<0.01$, '*': $p<0.05$). The diagonal in each set represents the distribution of f_3 values calculated for all reads (unfiltered), short reads <70 bp and long reads >100 bp. The figure was generated by R (v.3.5)[13] library ggplot2[14] in RStudio (v.1.3)[15].

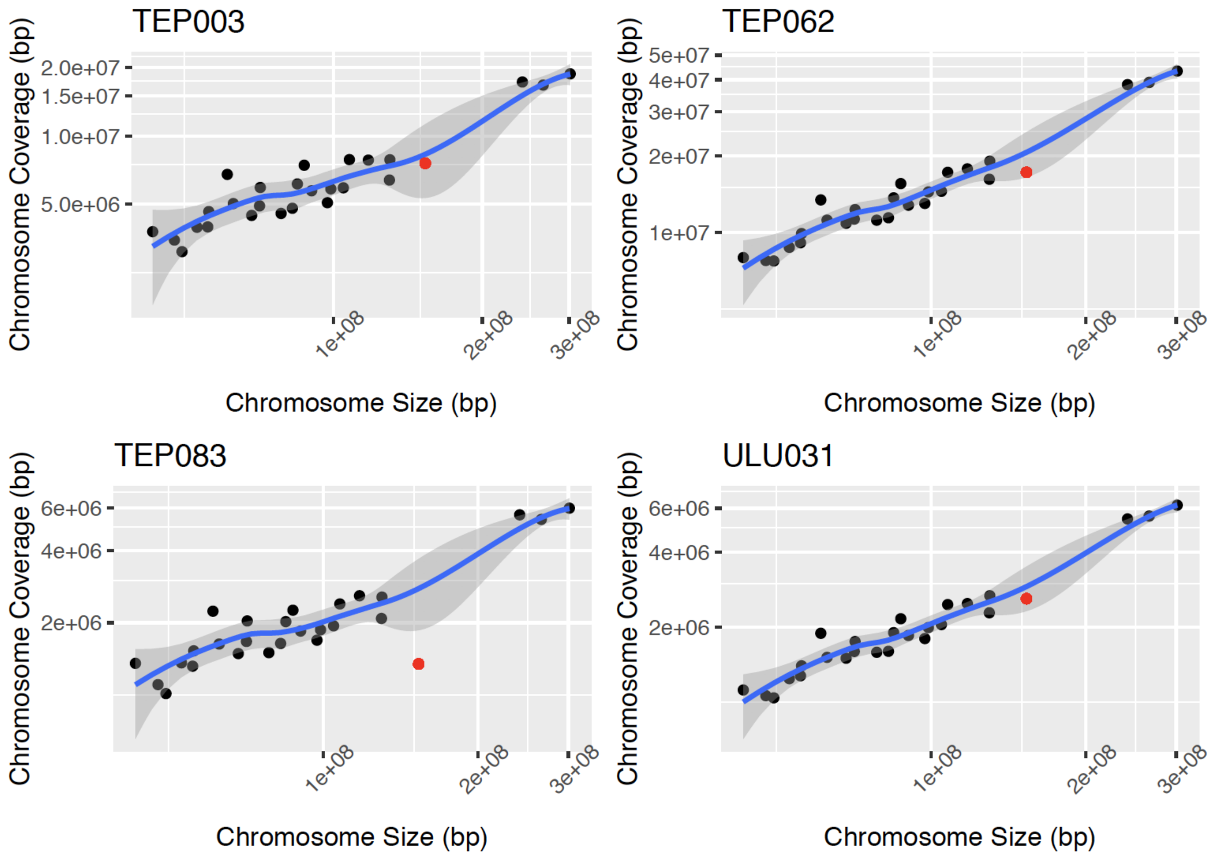

Supplementary Fig. 8: Molecular sex estimation of Anatolian Neolithic sheep individuals used in genomic analyses. Sex was estimated based on comparison of number of reads mapping to sheep chromosome X and autosomes, after duplicate removal and quality filtering. For each ancient individual, for all chromosomes, the number of reads (y-axis) were plotted against the size of the chromosome (x-axis). Black dots represent autosomes while chromosome X is depicted as a red dot. In each plot, 95% confidence intervals were constructed using a t-based approximation by 'loess' function of R (v.3.5)[13]. Note that the regression model was constructed only using autosomal data, and chromosome X only used for prediction.

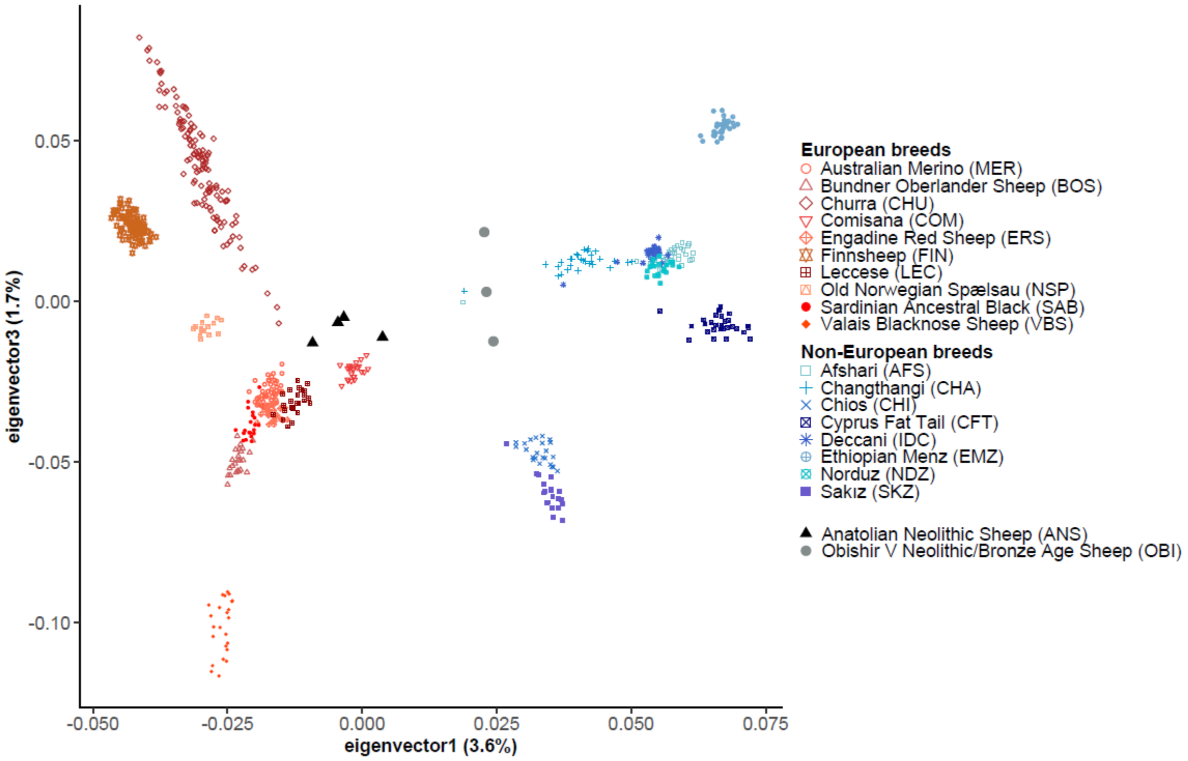

Supplementary Fig. 9: Genomic variation of modern breeds, Anatolian Neolithic sheep and Obishir V[2]. The first and third components of the PCA were calculated using genotypes of 18 modern sheep breeds. The four Anatolian Neolithic sheep individuals' genotypes (black triangles) and three Obishir V sheep individuals' genotypes (grey circles) were projected onto these two components. Parentheses indicate the proportion of variance explained by each principal component.

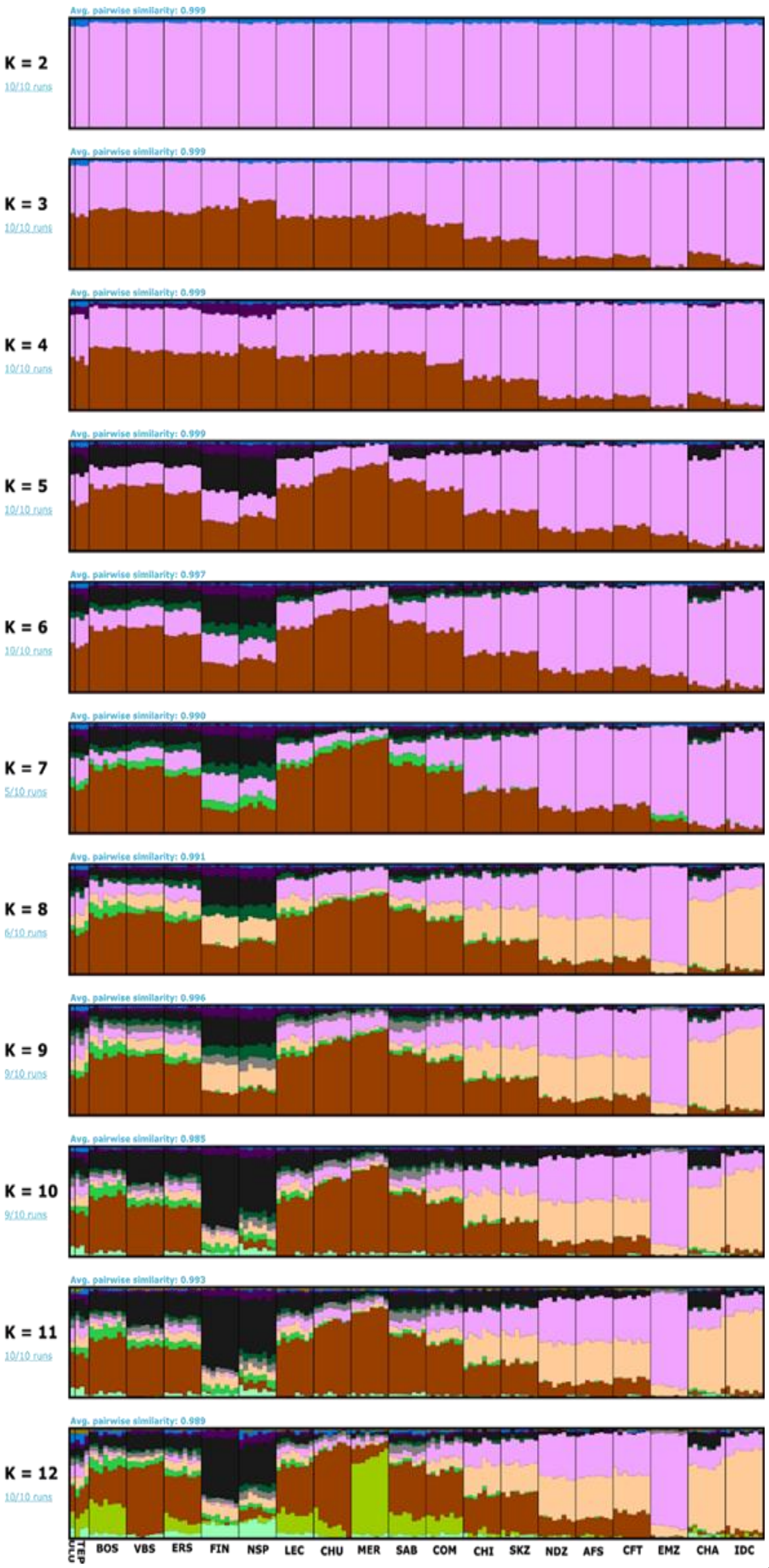

Supplementary Fig. 10: The unsupervised ADMIXTURE[16] analysis of ancient Anatolian Neolithic sheep and modern sheep breeds. Eight individuals were randomly chosen from each modern breed. The ANS genotypes were projected onto the calculated components. The components were ordered and colored using the Pong[17]. Origins of modern breeds are as follow: European (BOS, VBS, ERS, FIN, NSP, LEC, CHU, MER, SAB, COM) Non-European (CHI, SKZ, NDZ, AFS, CFT, CHA, IDC, EMZ).

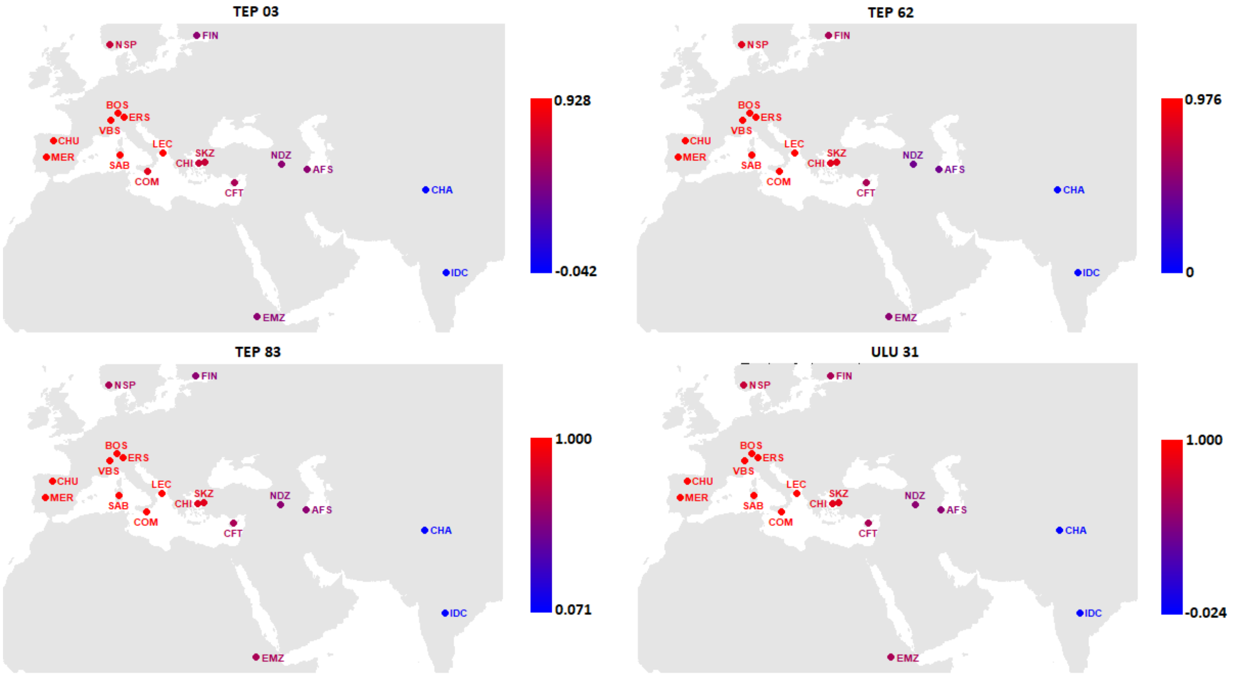

Supplementary Fig. 11: Correlation coefficients for 18 breeds, calculated on ADMIXTURE[16] components. We calculated ancestry component values from ADMIXTURE analysis with K=8 (Supplementary Figure 10), for each of n=18 modern-day breeds, using the average ancestry component value across each individual within the sample. We then calculated the Spearman correlation coefficient between the average ancestry component values of each modern-day breed and those of each ANS individual. Red and blue represent high and low correlation, respectively. All calculations and data visualizations were done using the RStudio (v.1.3)[15].

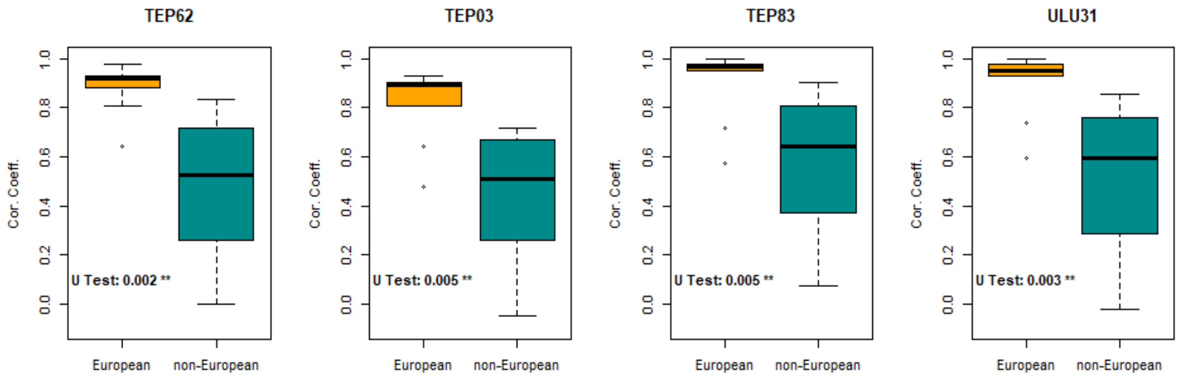

Supplementary Fig. 12: ANS affinity to European and non-European breeds based on ADMIXTURE[16] ancestry components. The boxplots show the distributions of Spearman correlation coefficients calculated between the weights of ADMIXTURE (K=8) ancestry components of each ANS sheep and those of European breeds (orange) (MER, BOS, CHU, COM, ERS, FIN, LEC, NSP, SAB, VBS) and non-European breeds (dark cyan) (AFS, CHA, CHI, CFT, IDC, EMZ, NDZ, SKZ), with total n=80 and n=64, respectively. The Mann-Whitney U test was performed to assess the difference between the two correlation coefficient distributions. P-values are shown in the inset. All calculations and data visualizations were done using the RStudio (v.1.3)[15]. See also Supplementary Fig. 10 and Supplementary Fig. 11.

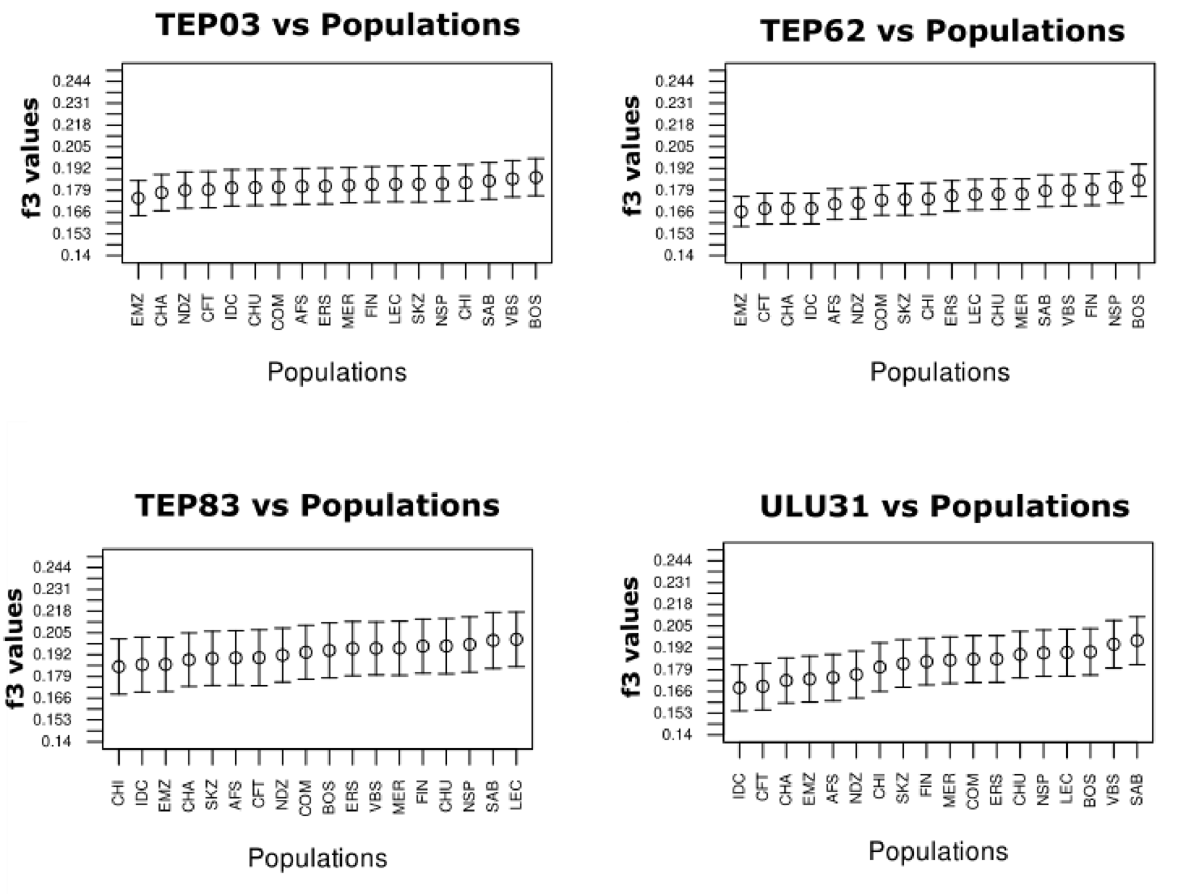

Supplementary Fig. 13: Outgroup f_3-statistics between each ANS individual and modern-day breeds. Outgroup f_3-statistics were calculated as f_3*(Argali; ANS, Modern)* (Supplementary Data 6 Sheet B). The breed name abbreviations are listed in Supplementary Table 3. Error bars indicate +/- 1 standard error from the mean.

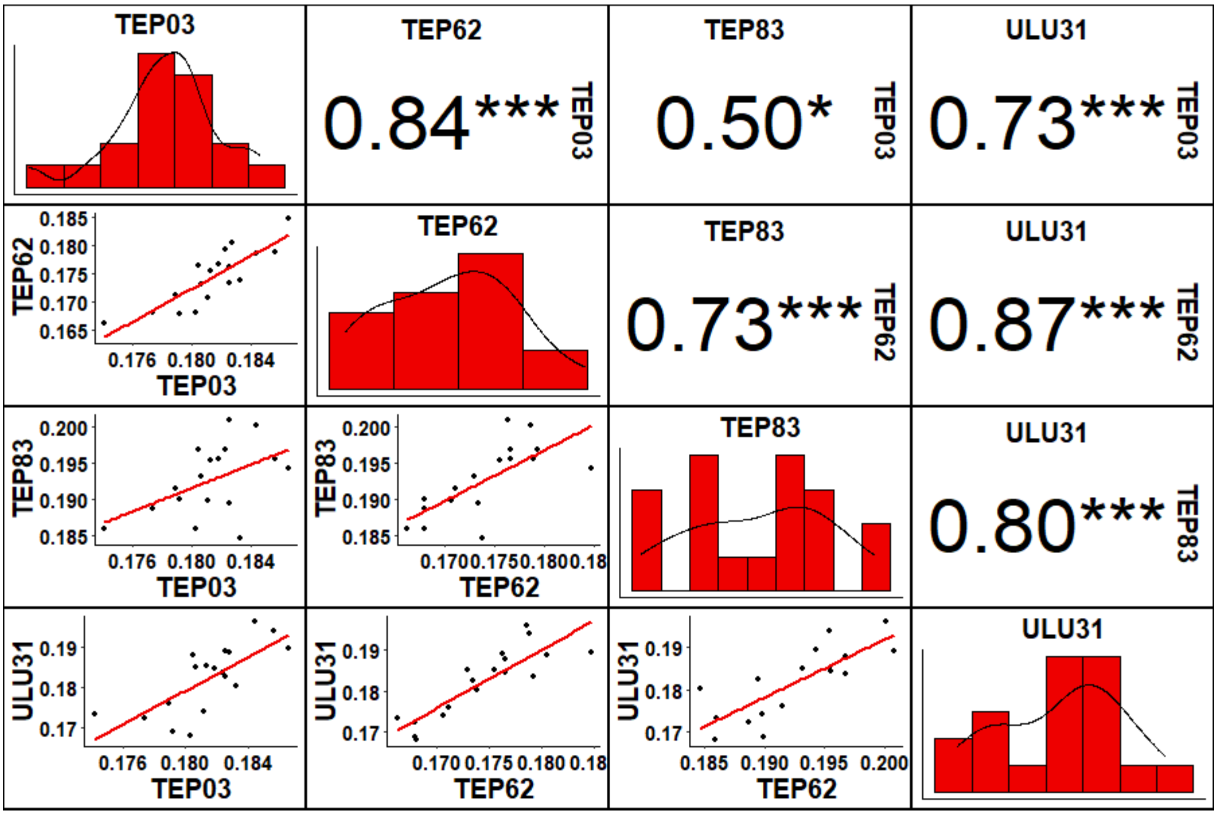

Supplementary Fig. 14: Comparison of modern-day vs. ANS sheep outgroup f_3-statistics across ANS individuals. In the scatter plots presented in the lower triangle of each panel, each point represents an f_3 value calculated between an ANS individual (TEP03, TEP62, TEP83 and ULU31) and a modern breed, calculated as $f_3(Argali; ANS_i, Modern)$, and the two axes represent f_3 values for two different ANS individuals. For example, the top leftmost panel is TEP03 f_3 values vs TEP62 f_3 values, for the same modern breeds. The red line represents linear regression. The upper triangle panels show the Spearman correlation coefficients for the corresponding scatter plots; asterisks indicate significance of the Spearman test ('***': p<0.001, '**': p<0.01, '*': p<0.05). The diagonal represents the distribution of f_3 values per individual, indicated in the inset. The figure was generated by R (v.3.5)[13] library ggplot2[14] in RStudio (v.1.3)[15].

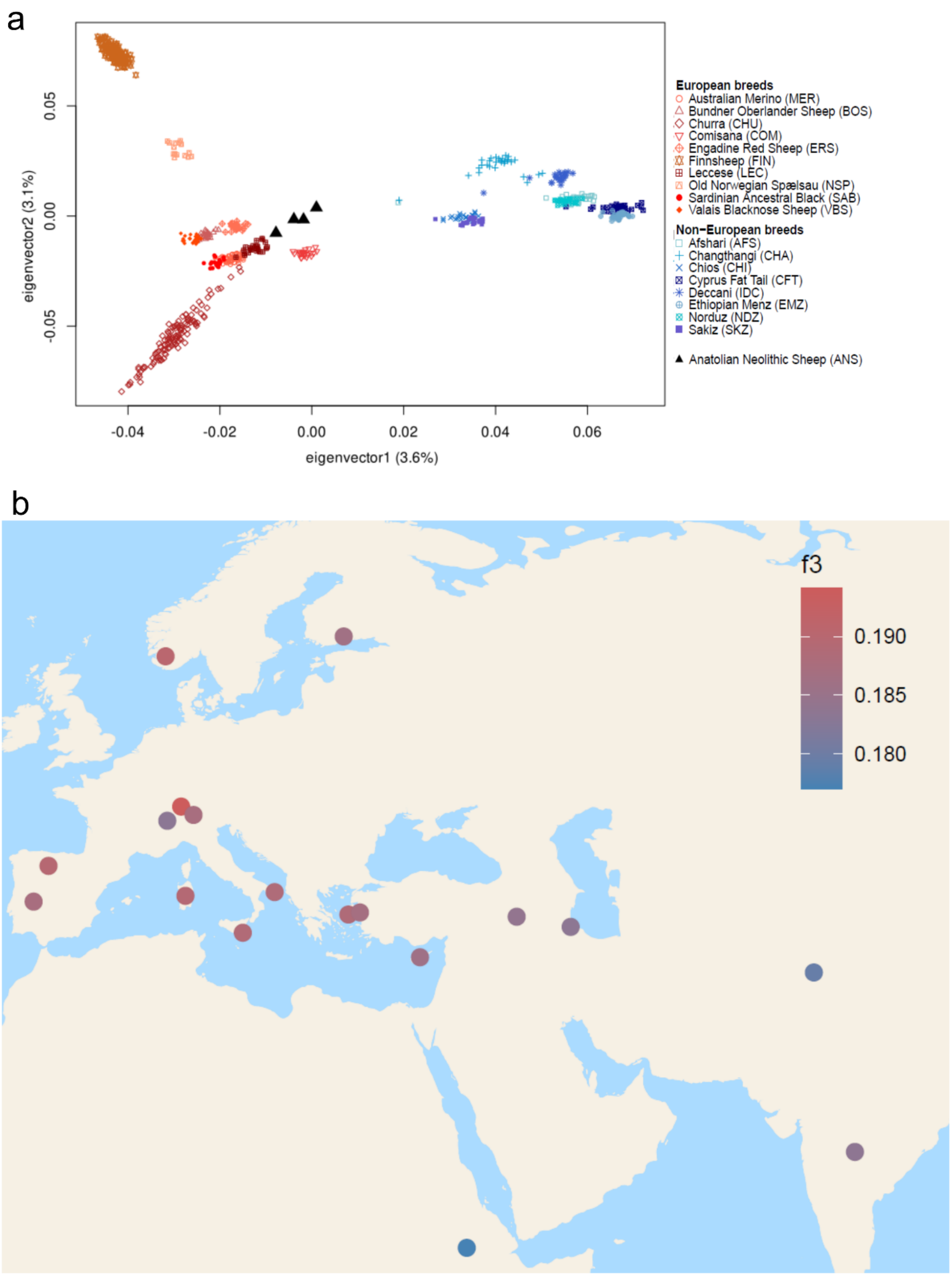

Supplementary Fig. 15: Principal components analysis and outgroup f_3 plots of Anatolian Neolithic sheep and modern sheep breeds calculated using transversion SNPs only. The panels a) and b) were produced in the same way as Fig. 3 and Fig. 4. Note that transversion type SNPs are not expected to be confounded by postmortem damage.

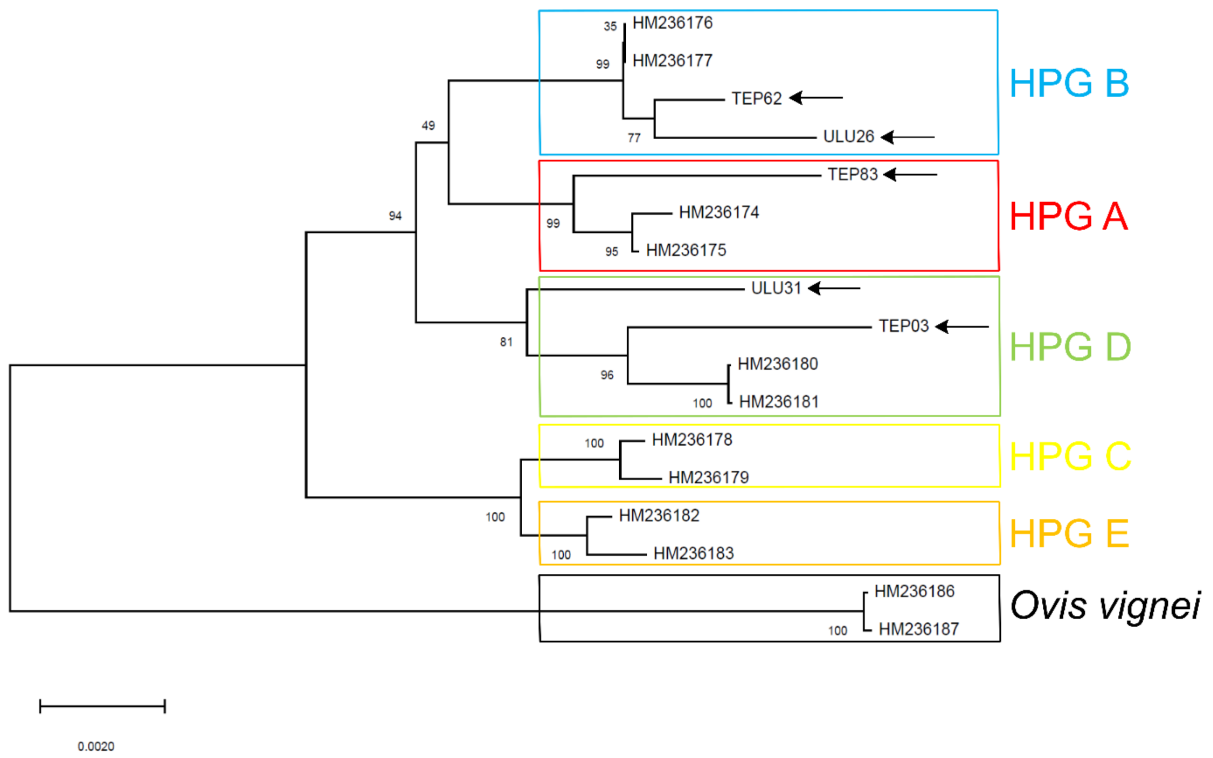

Supplementary Fig. 16: NJ tree of ancient and modern-day sheep mitogenomes. We joined eight ancient mitogenomes, 10 modern-day mitogenomes chosen to represent each haplogroup by two samples[18], and *Ovis vignei* as outgroup[18]. We then constructed a neighbor joining (NJ) tree on the combined data using MEGA-X[19]. Mitogenomes shown by solid arrows belong to Anatolian Neolithic Sheep samples presented in this study (n=5 for which we could produce sufficient genome data). The NJ tree constructed using 311 modern samples and 5 ancient samples is given in Supplementary File 9 in newick format.

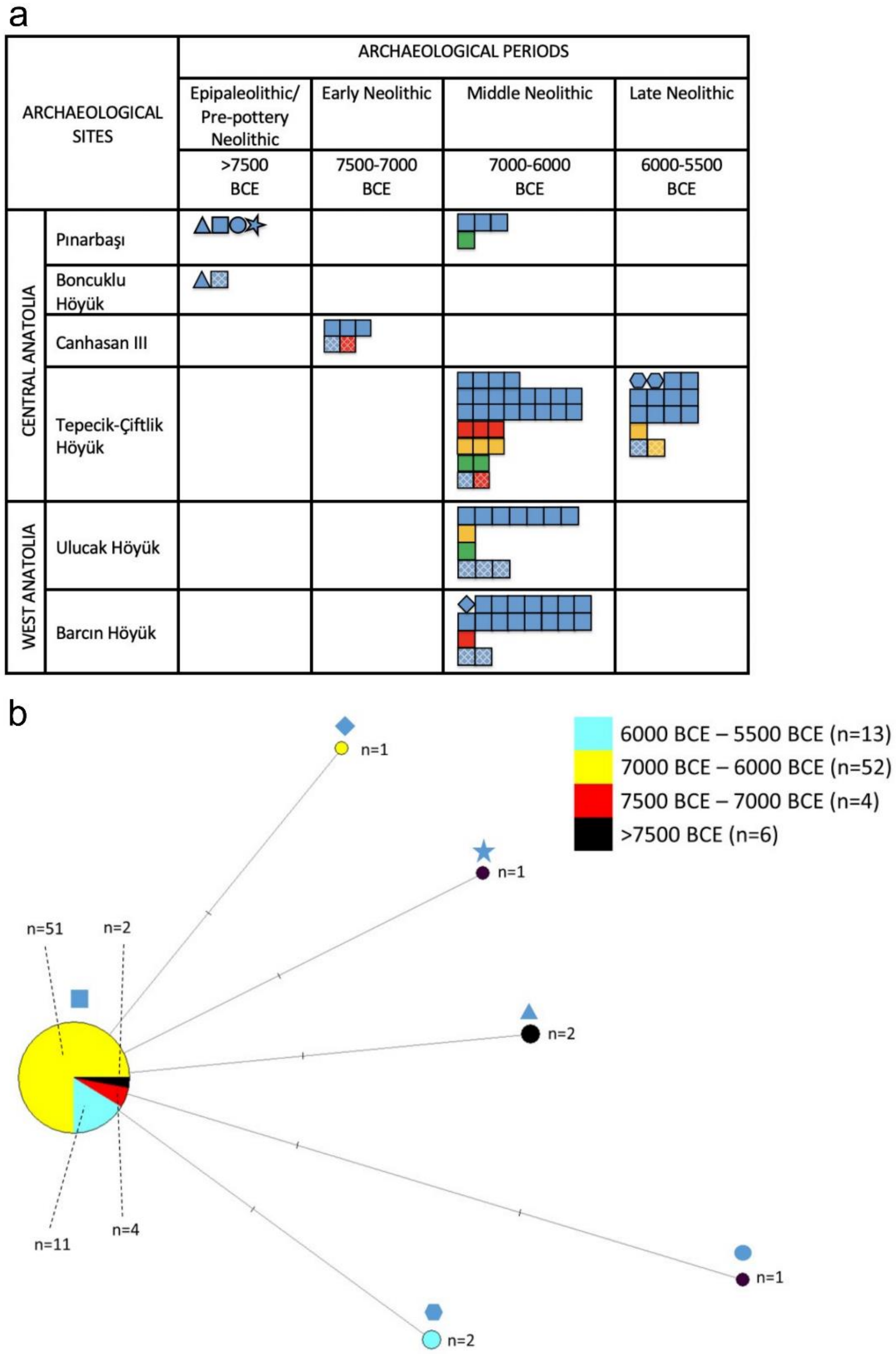

Supplementary Fig. 17: Mitochondrial haplogroup and haplotype diversity in Epipaleolithic to Late Neolithic ancient sheep from Anatolia. (a) The table shows the distribution of different HPGs, indicated by colors, and different HPG B haplotypes, indicated by unique geometric shapes. Color coding is as follows: HPG A (red), HPG B (blue), HPG C (yellow), HPG D (green) and HPG E (orange). Individuals supported by a single amplified sequence are shown with hatched pattern. (b) Median-joining tree of the observed haplotypes from Epipaleolithic to Late Neolithic within HPG B individuals based on the 144-bp mtDNA fragment. Each geometric shape in blue on top of the nodes corresponds to a different haplotype, also indicated in Fig. 17a. We should emphasize that, 24 of the 25 modern HPG B individuals from Akkaraman, Sakız and Kıvırcık (not shown in this figure) fall within the largest node (represented with blue square) while one individual forms a new branch with a single mutation difference from the most common haplotype.

Supplementary Note 1

<u>Background on Ovis species relevant to the study, domestication center, mitochondrial DNA haplogroups (HPGs), spatial diversity patterns of mtDNA HPGs, justification of using 144 bp part of CR for HPG determination in ancient sheep</u>

Ovis distribution and domestication

Wild sheep, Asiatic mouflon (*Ovis orientalis*, abbreviated as *O.o.*) is accepted as the ancestor of domestic sheep[20]. The native distribution of *O.o* is not well known. As is attested in archaeological complexes, the span of its distribution is from western and southwestern Anatolia to central Anatolia, non-coastal parts of Levant, southeast Anatolia, Zagros mountains and central Iran[21]. Within this wide range of *O.o.* distribution, the sheep domestication center was proposed to be the area spanning from eastern central Anatolia to northwestern Iran[22–24]. It is generally accepted that domestication of an animal species could occur multiple times within a domestication center[24], and there might be other domestication areas, which have not yet been identified for sheep. Furthermore, introgression(s) from wild population(s) might have differentiated domestic populations. Domestication and introgression are both considered as parts of the episodes of a domestication process. Studies unfolding the domestication process of sheep are in their infancy.

Wild sheep were not present in Europe and Africa in recent geographic times[21]. Sheep entered Europe and Africa only as domestic animals, after being domesticated in northern parts of Fertile Crescent[24]. Another wild sheep is Urial (*O.vignei*) from western central Asia, which is the eastern neighbor of *O.o* and their distributions overlap in the eastern Iran, whereas Argali (*O.ammon*) from central Asia is distributed on the east of Urial sheep range. For the statistical tests Argali was chosen as an outgroup in the present study. All of these wild sheep species can hybridize[8,25–27]. After domestication there may have been introgression from wild sheep to domestic sheep during its spread throughout Anatolia and Asia. However, we can exclude introgression from the west, because wild Ovis are thought to have been absent in Europe by the early Holocene[21,25].

Past and present mtDNA haplogroup diversity in domestic sheep

The mtDNA control region (CR) has been widely employed in the earlier modern domestic sheep studies to investigate the evolutionary history of domestic sheep. Five haplogroups, from HPG A to E, have been observed in sheep[28]. HPG B is currently the most prominent (~89%) and widely distributed HPG in the world. HPG A, the second most frequent HPG, occurs generally more frequently than 30% in the Asian breeds and highly frequently (~77%) in India, however, it occurs less frequently than 10% in Europe. HPG C, the third common HPG, is relatively frequent (~20%) around the Caspian Sea, in the Middle East and northern China[4]. Frequency of each HPG in some selected regions from Europe and Asia are given in Supplementary Table 6. Only 1% of the sheep exhibit HPGs E and D[4]. All five HPGs are observed only in the Middle East region and among present-day native breeds of Turkey[18].

Ancient mtDNA studies on ancient sheep conducted to date suggest that by around 6,000-5,500 BCE HPG A frequency was already high in regions to the east of Turkey. Specifically, 60% of 10 sequences from Georgia[29] (5,300-5,600 BCE), 47% of 15 sequences from Iran (c.6,000 BCE)[30] and 60% of five sequences from China (c.6,000 BCE)[31] exhibited haplogroup A. In a recent study on sheep samples from Obishir V, Kyrgyzstan, two Neolithic individuals and one Bronze Age individual were found to carry HPG A[2]. In our dataset such high frequencies of haplogroup A were not seen, either in central or in west Anatolia, between the Epipaleolithic to 5,500 BCE. This suggests that there could have been another domestication episode involving sheep with high HPG A frequency in some other areas of the native distribution of wild sheep, for instance around the Zagros mountains and or the Caucasus. Intense wild introgression by a population with high HPG A frequencies in some region of Asia is also possible. Human archaeogenome studies revealed that there were migrations from Levant and Iran/Caucasus to Anatolia during 7,500 BCE-6,500 BCE[32,33]. Thus, it can be anticipated that as well as A, lineages E and perhaps D might have arrived from south and east of the sheep domestication center.

The rationale for analyzing mtDNA

Mitochondrial DNA acts as a single non recombining gene, and therefore the information it provides cannot completely reflect complex demographic histories (for instance see Keis et al.[34], Larson and Burger[35], Paijmans et al.[36]). Hence, nuclear genome studies are necessary to fully unfold the recent demographic history of sheep. Nevertheless, we anticipated low DNA preservation and a low success rate in retrieving sufficient genetic data from Anatolian Neolithic sheep, because of exposure to many DNA degrading factors, especially high heat during the cooking. Due to the presence of hundreds of copies of mtDNA in a single cell, small fragments of mtDNA can be more easily available when genomic DNA is largely lost. Indeed, our success rate in obtaining >1% endogenous DNA was low (4/29, ~14%), whereas our mtDNA retrieval rates were higher (20-60%). The large mtDNA sample thus obtained allowed a number of observations made based on nuclear data to be confirmed.

Accuracy of haplogroup assignment based on the 144 bp CR fragment

When mtDNA is used in phylogenetic studies, it is preferable to examine the whole mitogenome sequence rather than its subparts[18]. However, Sanger sequencing-based mitogenome analyses in ancient samples needs labor intensive PCR amplifications of many overlapping short (~150 bp) regions. Further, in an aDNA study, it is highly unlikely to amplify all regions. Additionally, to detect post-mortem changes (e.g. C to T transitions) every region must be amplified at least twice. These obstacles can result in low success rates despite the allocation of a large amount of effort and cost. Accordingly, we chose to utilize the most informative single region of the mtDNA, i.e., the control region (CR)[18], and focus on a short fragment that could be amplified in a single experiment.

We chose a 144 bp long fragment of the CR that harbors diagnostic sites to identify five haplogroups of domestic sheep[8,37]. We checked the power of this 144 bp region to accurately identify haplogroups. For this, we studied haplogroup assignment across 311 modern sheep mitogenomes[4,18,45–50,20,38–44]. We constructed a neighbor joining (NJ) tree with 1,000 bootstraps,

assigned haplogroups based on their cluster on the NJ tree (Supplementary Data 9, Supplementary Fig. 16). The same 311 modern sheep were also assigned haplogroups based on the 144 bp-long fragments, and the results compared to mitogenome-based assignments. Using the latter as reference, we estimated the accuracy of haplogroup assignments based on the 144 bp fragment (Supplementary Data 10). Among the modern samples, on the other hand, HPG assignment of 14 individuals were inconsistent: 0.63% of the individuals were assigned HPG C based on 144 bp and were assigned to HPG A based on NJ, 0.95% of the individuals were assigned HPG D based on 144 bp and were assigned to HPG C based on NJ, 0.63% of the individuals were assigned HPG B based on 144 bp and were assigned to HPG A based on NJ and 0.95% of the individuals were assigned HPG A based on 144 bp and were assigned to HPG B based on NJ. Meanwhile 0.63% of the individuals could not be assigned to any HPG based on 144 bp fragment but they were assigned to HPG A based on NJ.

Overall, haplogroup assignment accuracy using the 144 bp long fragment was estimated as 95.6% in this sample. Inconsistencies appear mainly due to convergent transitions in this region, which may not be surprising given the known high mutation rates in the mtDNA control region.

We further studied haplogroup assignment accuracy of Sanger-sequenced 144 bp fragments using data from five ANS individuals with sufficient high-throughput sequencing mitochondrial DNA coverage of >1,500 bp (ULU26, in addition to the four ANS individuals presented in Table 1; see Supplemental Table 2). The assignments of TEP83, TEP62, ULU26 were consistent between Sanger-sequenced 144-bp fragments and mitogenome-based assignments. TEP03 and ULU31 were assigned to haplogroup B based on the 144 bp fragment data but assigned to haplogroup D using their mitogenome data (Supplementary Figure 16). The reason was a C->T transition at the single diagnostic position that differentiates haplogroups D and B. We could rule out postmortem damage-induced transitions, because (a) all Sanger-sequenced fragments carried the same allele, (b) NGS data contained the same allele, (c) the position was 20 bp inside the fragment (whereas postmortem damage is expected to occur near fragment ends).

Accordingly, the haplogroup assignment inconsistency we observe for TEP03 and ULU31 appears to arise from a genuine convergent transition. This would be alike convergent mutations identified among modern mitogenomes above. We thus do not expect the overall occurrence of haplogroup misassignment in our ancient sample to be necessarily higher than the misassignment estimate (approximately 5%) based on n=311 modern mitogenomes.

Supplementary Note 2

Sample informations and archaeological contexts

Pınarbaşı (by Douglas Baird)

Pınarbaşı is located on the eastern edge of the south-west Konya basin, 24.5 km south-east of Çatalhöyük and 30 km south-east of Boncuklu, on the tip of a limestone ridge projecting from the foot of the Karadağ volcanic massive. The site was excavated under the direction of Prof. Trevor Watkins of Edinburgh University in 1994 and 1995 and by Professor Douglas Baird of University of Liverpool in an excavation and study project running 2003-2006. The site runs from a series of cliffs on the western edge of the limestone ridge, over the slopes in front of the ridge and onto a small mound projecting into the Hotamış lake basin. The zooarchaeological studies were carried out by Dr Denise Carruthers and Dr Caroline Middleton.

There are four main prehistoric periods of occupation at the site, three of which have provided samples for the present study. The earliest occupation so far detected at Pınarbaşı is found only in a rock-shelter next to the cliffs and is dated by C14 to the Late Glacial/Epipalaeolithic from c. 14,000-11,000 cal BCE[51] and was excavated in Area B[51]. The second period represented at the site dates from c. 9,600-7,800 cal BCE in the early Holocene[52] and is found only on the mound projecting into the Hotamış basin and was excavated in Areas A, C and D[52,53]. The third main period, a Late Neolithic occupation, dating c. 6,500-6,000 cal BCE is found only in the rockshelter, was excavated in Area B, overlying the Epipalaeolithic deposits and was separated from them by a substantial amount of limestone debris. The fourth main prehistoric period, which has not contributed any samples to this study, is represented by Late Chalcolithic-Early Bronze Age occupation; covers the whole site and was excavated in areas A, C and D.

The Epipalaeolithic saw probably short episodes of seasonal occupation by small mobile foraging groups, certainly including the summer and autumn seasons, repeated over millennia, including burials as well as residential soujourns[51]. Fishing, fowling and hunting were key subsistence activities when this group camped at Pınarbaşı. Mammal hunting practices included a significant focus on caprines, the most common of the larger mammal species (c. 46% of the Number of Identified Specimens- NISP) in the zooarchaeological assemblage, presumably available on the slopes of the Karadağ to the east of the site, and amongst which the sheep:goat ratio is 3:2[51]. There is no evidence in their anatomical morphology or the C and N isotopes to suggest management of the caprines[11].

The three Epipalaeolithic samples in this study derive from several different contexts spanning most of the sequence of Epipalaeolithic occupation deposits. The samples come from different phases in the stratigraphic sequence that spans 3,000 years minimum. There is little evidence that Epipalaeolithic activity at the site involved excavation of deep features and therefore redeposition is likely to have been a minimal part of the formation processes on the site. The three HPG B samples in this study were deposited in contexts separated by centuries or millennia and it is very unlikely that they derive from the same individual. These three samples belong to three different haplotypes: shown by a square, star and circle (Supplementary Fig. 17) including the haplotype that becomes predominant (square) in the earliest probably

domestic caprines after 7,500 cal BCE at Canhasan III and this haplotype was therefore locally present amongst sheep populations c. 6,000 years before evidence of domestication. This allows the possibility of a local domestication event in central Anatolia and more specifically the Konya plain area. However, we should emphasize that our argument is based on contextually dated (not C14 dated) samples. Also note that throughout the discussion sequences of individuals with two or more readings were considered. The early Holocene occupation at the site dates from c. 9,600-7,800 cal BCE[52]. This community was resident at the site on a multi-seasonal basis[52]. Sunken curvilinear plastered structures with wattle and daub superstructures were present along with significant numbers of burials. Together this evidence suggests long term investment in and commitment to the Pınarbaşı location as a residential area[52]. Baird has thus suggested this as a sedentarising behavior. This community engaged in foraging practices with no evidence of cultivation and a focus on gathering local nuts/fruits, wild almond, terebinth and hackberry and the hunting of larger mammals, especially aurochs, accompanied by some fowling and fishing. Caprines were morphologically wild and were thus also regularly hunted, c. 27% NISP[52]. The sheep:goat ratio amongst the caprines was 8.5:1. As during the Epipalaeolithic sheep would have been quite locally available particularly on the foothills of the Karadağ to the east of the site. Study of the caprine isotopes indicate a diet very similar to that of the Epipalaeolithic caprines lacking C4 plants in the diet and eating plants low in $\delta^{15}N$ suggesting a diet not impacted by humans[54].

Sample PB2 yielded sufficient aDNA for the purposes of this study; it derived from general midden deposits in the sequence in Area A, dating to the 8,500-7,800 cal BCE phases of occupation of the site. This is also HPG B and the same haplotype (triangle) as the single broadly contemporary Boncuklu samples. The haplotype of this group was not identified in the Epipalaeolithic sample, which may be not surprising given the small sample size. This haplotype is also not seen in the probably earliest managed sheep after 7,500 cal BCE. Assuming that this change indicates genetic bottlenecks associated with domestication, this observation would indicate that such bottlenecks may not have commenced before 7,800 cal BCE.

The third period of occupation at Pınarbaşı was located only in the rock shelter area and dated to the Late Neolithic c. 6,500-6,000 cal BCE[55]. This was contemporary with the later levels at Çatalhöyük East. The occupations had a strongly seasonal nature, with spring particularly well represented, but probably some autumn visits as well. Morphologically wild cattle and equids are attested in the assemblage, but the highest proportions of larger mammals are caprines, NISP 72.5%, morphologically domesticated and kept close to the occupation area judging by large quantities of perinatal remains and herbivore dung in deposits on the site[55,56]. Amongst the caprines the sheep:goat ratio is 168:1[55], showing a massive preponderance of sheep in the flocks and a major shift compared to ratios in the earlier assemblages. The C and N isotopes also strongly indicate these caprines are herded, given their generally elevated $\delta^{15}N$ and significant proportion of individuals consuming C4 plants, with isotope signatures close to contemporary Çatalhöyük[54,55].

Four samples in this study derived from these Late Neolithic occupation phases at the site. These come from Phase E I early in the use of a Late Neolithic structure in the rock shelter

area[55]. It is impossible to rule out that 2 or 3 of these might have derived from a single individual since they are all from the same context. However, this context represents a deep build-up of ashes including several episodes of dumping of deposits into the structure. The context likely accumulated over a period of a minimum of weeks and likely years. The deposits contained large numbers, including immature and adult caprines. Given these indicators it is likely that these samples derived from different individuals. We thus see the dominance of HPG B haplotype that seems to predominate amongst the domesticated sheep of Neolithic central Anatolia, and the addition of haplogroup D, not seen in the wild population sample from the area. It is attractive to speculate that haplogroup D could have been introduced to the area by c. 6500 cal BCE. If these results are supported by larger sample sizes in future studies, they could be interpreted as evidence of movement of sheep between communities during the Neolithic, through exchange, pastoralism and/or accompanying the movement of people into the Konya plain.

Boncuklu Höyük (by Douglas Baird**)**

Boncuklu is an archaeological mound in the middle of the south-west Konya basin in south central Anatolia. It is located 9.5 km north of the site of Çatalhöyük. Excavations have been directed by Professor Douglas Baird from the University of Liverpool since 2006 and co-directed with Baird by Professor Andrew Fairbairn, University of Queensland and Dr Gökhan Mustafaoğlu, Ankara Haci Bayram Veli University, since 2011. The zooarchaeological studies were carried out for most of this period by Dr Louise Martin and Dr Caroline Middleton. Evidence for occupation spans 8,300-7,600 cal BCE. The site contains a series of sub-oval mudbrick structures, some with sub-floor burials, as well as burials in open areas. Buildings have standard domestic features and notably the space is divided into ‘clean’ and ‘dirty’ areas. This evidence combined with evidence for occupation of the site in all seasons over a prolonged period strongly suggests an early sedentary community[52]. Adoption of cultivation by indigenous foragers[32] is suggested at Boncuklu and likely by 8,300 cal BCE[53]. Cultivated wheat and legumes probably made a limited contribution to the diet. Cultivated plants may have been introduced through interactions with areas to the south and east in the 9th millennium cal BCE. Morphologically wild cattle - aurochs, and probably wild, boar represent the main hunted species. In contrast to Pınarbaşı, caprines represent a very small element of the faunal NISP, c. 4%, with a sheep:goat ratio of c 1:1.33[52]. This is not surprising as the closest wild caprine natural habitats would be the Bozdağ c. min 15 km to the north. However, C and N isotopes, with some individuals with elevated δ^{15}N suggest some human effect on caprine diet[54] and along with herbivore dung on the site[56] hint at early management. In that context it is notable that an aDNA sample, from the site is the same haplogroup (triangle) as broadly contemporary Pınarbaşı, unlike the somewhat later probably early domestic caprines, with little evidence of the commencement of genetic bottlenecking indicated by the predominance of the haplotype seen at Canhasan III and Pınarbaşı Late Neolithic. Given Middleton[54]’s C and N isotope results this may well point to management definitively preceding the development of the genetic domestication process. In addition, it is interesting that a haplotype that was apparently not uncommon and widespread in the 9th millennium cal BCE Konya plain was not caught up in the bottleneck of domestication.

Canhasan III (by Douglas Baird)

Canhasan III is located on the Karaman alluvial fan south-east of the volcanic massif of Karadağ in the Konya Plain and c. 90 km South-east of Çatalhöyük and was excavated in 1969-70 by David French[57], and is dated to c. 7,400–7,100 cal BCE[58]. At the request of Dr French, Prof. Douglas Baird is overseeing study and analysis of material and information from the site to more substantive publication. French excavated a 20x30 m area by surface scraping in which he exposed an extensive dense set of abutting rectangular structures similar to sites like Aşıklı and Çatalhöyük. He also excavated in a much smaller area, in square 49L, a deep sounding to 6.75 m which stopped at the water table. The plant remains indicate the exploitation of wild species as well as cultivated cereals. The animal remains, which are subject to ongoing study, indicate an important presence of cattle and caprines. The domestication status of these species is unclear[59]. A recent study of caprine diets through C and N isotopes by Middleton[54] strongly indicated that a high proportion of caprines consumed C4 plants and most individuals showed high $\delta^{15}N$, like the later Çatalhöyük East morphologically domestic caprines, were thus probably herded.

The three samples within this aDNA study were from both the wider scraped area and the deep sounding. They date to different phases and parts of the site and thus based on the stratigraphic and contextual record are unlikely to be from the same individual(s). They are all of the HPG B and the sequence that predominates (square) in slightly later domestic sheep flocks and this supports the evidence of C and N isotopes that the Canhasan III sheep (and probably therefore goats) were herded, with high degrees of management and, in addition, that this may have resulted in genetic bottlenecks. This evidence also ties down the appearance of clearly herded sheep on the Konya Plain to the period between c. 7,800 and 7,300 cal BCE.

Tepecik-Çiftlik (by Erhan Bıçakçı and Yasin Gökhan Çakan)

Tepecik-Çiftlik mound is located in the Çiftlik district of Niğde province which resides in the Volcanic Cappadocia region of the Central Anatolian Plateau. The mound has an area of 6 hectares and is 9.6 meters above the plains it is located. Although the mound was excavated 7.3 meters below the ground, the rock soil has not been reached yet[60]. Excavations carried out in the mound since the year 2,000 pointed out that from the end of the 8th millennium BCE to the beginning of the 6th millennium BCE the settlement was occupied by an agriculturalist community. Alongside the farming, the community continued hunting and gathering the rich resources of the region. Significant changes in the architecture and settlement layout could be observed during the 1,200-year period that straddled from the early Pottery Neolithic towards the end of early Chalcolithic[61]. Almost every level has a different layout. Although the buildings in different levels were built with similar techniques, they differ from each other in terms of space perception and settlement pattern. Sheep and goat were the highly dominant animal species in the settlement. They were followed by cattle, equids and red deer, respectively. Additionally, the other species detected among the animal remains were roe deer, fallow deer, canids, rabbit, bear, rodents, pig and some bird species. Domesticated sheep, goat and cattle were observed after the first half of the 7th millennium BCE. However, hunting wild animals was also part of the subsistence[62,63]. The oldest known pottery in the Volcanic Cappadocia

region is found in Tepecik-Çiftlik[64]. The continuous stratification in the mound permits to follow both technological and typological development from the first time the pottery appeared to the end of the early Chalcolithic period. The obsidian production areas within the settlement emphasizes the significance of processing and the use of this raw material for the community[65]. Almost all types of the arrow and spearheads known from the contemporaneous Central Anatolian settlements are also found in Tepecik-Çiftlik. It is known that the obsidian blades and cores produced in Göllüdağ workshops made their way to the settlements in faraway regions such as the Levant, Northern Syria and Cyprus since the earliest phases of the Neolithic period[66]. Networks established between the obsidian workshops and settlements could be regarded as the most concrete indication of the mutual transfer of knowledge and technology between these regions. It should be noted that the Tepecik-Çiftlik people were aware of the developments taking place in the workshops immediately next to the settlement[67]. Archaeological discoveries in Tepecik-Çiftlik indicate a community that could make good use of the natural resources around it and that retains close relationships with the nearby as well as the distant regions.

Most of the 45 Tepecik-Çiftlik Höyük sheep samples analyzed in this study were obtained from open areas and to a lesser extent from pit fillings. None of them belong to the interior fillings. Each sample analyzed in this study was examined in terms of its context, taking into account the collection unit numbers specified in Supplementary Table 1 to assess whether any two or more samples could belong to the same individual. As a result of the examination, the samples numbered 16-60 in Supplementary Table 1 were divided into eight different groups according to their archaeological context. It is unlikely that bones sampled from different contexts can belong to the same individual in terms of archaeological stratigraphy rules. Although there is a chance of sampling the same animal's bones and registering them as different individuals, we do not think it is the case here, because the majority of our samples were found in open area fillings which are complex fillings that have been formed throughout many years. The bone fragments found in these were usually individual fragments that were dispersed and isolated. The archaeological context of Tepecik-Çiftlik Höyük samples numbered 16-60 in Table 1 are as follows: samples 16-19 were obtained from the large pit filling with east-west extension identified in the upper phase of Level 2; samples 20-26 were unearthed from the yellow-colored hard structure, probably an open area fill, in the south of trench 15K in the upper phase of Level 2 whereas samples 31-32 unearthed from a pit filling belonging to the upper phase of Level 2. Samples 27 and 28 were obtained from the deepest fillings reached during the excavations; samples 29 and 30 are samples collected from the open area filling of the 2nd layer; samples 33-58 were obtained from the open area filling of layer 5/6. Most of this open area in the central part of the excavation area consists of rubbish-like fillings that can be described as "midden". We came across animal bones at every excavated point of this open area of approximately 20X20 meters. The fireplaces, waste areas and primary/secondary burials found in this area indicates that this part of the settlement was used very actively. Samples in this group were collected from various parts of the area. Samples 56-58 were unearthed from Level 7 of the deep trench in 16K; samples 59 and 60 were found in a votive pit from an open area at the transition of Level 3 and 4.

Ulucak Höyük (by Özlem Çevik and Evangelia Pişkin)

Ulucak Höyük, lies 25 km east of İzmir in West Central Turkey, is yet to be a key site in the region. The Neolithic occupation at the site is designated by Levels VI through IV and is dated between 6,850 cal BCE and 5,670 cal BCE[68]. A full-fledged agricultural economy with cereals and pulses and four-tiered herding system including sheep, goat, cattle and pig has been attested starting from the basal levels onwards[69]. The earliest occupational level is devoid of pottery and any other clay objects while sporadic occurrence of Melian obsidian in this level suggests contacts with the Aegean world were already established[70,71]. Pottery was adopted after 6,600/6,500 BCE although clay images including figurines and seals became an integral part of the material culture towards the end of the 7th millennium BCE[72,73]. The initial settlement (Level VI: 6,850-6,500 cal BCE) has so far been represented by two rectangular buildings with mud slab walls (Building 42 and 43) flanked by open spaces with fire installations. The floors and possibly the walls of these buildings were lime plastered and red painted. As the buildings appear to have been deliberately left clean, sheep bone samples were taken around the fire installations. Level V (6,500-6,000 cal BCE) with its five sub phases (a-e) is represented by post-framed and mud-slab buildings while both substantial mud-brick buildings on stone foundations and post-framed buildings are found in Level IV (6,000-5,670 cal BCE).

When collecting sheep bones from the Ulucak Höyük for aDNA analysis, it was first aimed to include only bones from the same element, the same side (left or right) which also had to meet satisfactory at least 70% of the published criteria for the identification/separation of sheep and goat[74,75]. Nevertheless, because the bones available at the time of selection were very fragmented and many of them burnt- hence unsuitable for aDNA analysis, it was not possible to follow this strict procedure. For this reason, various bones were included in the sample (still satisfying 70% of the criteria for identification). For safeguarding against sampling the same animal repeatedly, a combination of the following criteria was taken into account: element, side, age[76,77], osteometry[78], as well as archaeological information concerning the location and depth of the excavated context. According to these, from the 12 successfully analyzed sheep bones only 2 may belong to the same animal. These are the samples No 61 (ULU12) and No 70 (ULU9), both representing astragali. These bones have some difference in osteometric values, and they were excavated at different depths. Even so, these differences are judged to be small to guarantee that the two astragali do belong to different animals.

Barcın Höyük (by Rana Özbal and Fokke Gerritsen)

Barcın Höyük, a seventh millennium Neolithic site, is located in the Yenişehir Plain in the province of Bursa in northwestern Turkey[79]. Excavations here, directed by the Netherlands Institute in Turkey, yielded an uninterrupted Neolithic sequence from 6,600 cal BCE through 6,000 cal BCE. The lowest levels of the settlement represent the initial occupation by pioneer farmers who arrived in this region presumably from Central Anatolia[80]. These people brought with them domesticated stocks of plants and animals in a conscious effort to settle and colonize these regions[81,82]. It is possible that small numbers of foragers inhabited the region but the pioneers at Barcın Höyük maintained a fully Neolithic lifestyle[83]. They lived in rectangular timber structures organized in rows and surrounded by courtyard spaces[84]. The courtyards were

used for domestic activities and functioned as cemeteries especially for the adult population. Infants were buried beneath the floors of the houses while children were placed just outside the houses in the annexes or veranda spaces. The earliest inhabitants were accomplished potters though they did not initially rely on this technology for cooking or for storage and instead made use of indirect cooking techniques[85]. Pottery recipes were perfected through time; by 6,500 BCE the pots show expert burnishing and new tempers types were added to strengthen the vessels[84]. Domesticated cereals and pulses comprised the majority of the plant foods consumed although hazelnuts were also exploited[86]. With regards to domesticated animals, cattle and sheep dominate the assemblage[87]. Domestic goats too are found though in small numbers. Evidence for hunting and hunted animals is limited at Barcın Höyük although the faunal record included small numbers of wild boars, deer, hare. Dairying presumably from cattle but potentially also sheep was part of the diet from the earliest levels onwards[82].

The 19 successfully analyzed samples from Barcın Höyük have been collected from 16 different excavation contexts, representing 16 separate depositional episodes. They come from five different building levels, spread out over some four consecutive centuries (between 6,500 and 6,100 cal BCE). Although it cannot be ruled out completely, it is unlikely that skeletal remains from an individual animal became dispersed over separate depositional contexts. In three cases, two samples (BH3 and BH8, BH32 and BH41, BH15 and BH19) were collected from the same excavation context. It is conceivable that these sample pairs represent single animals, but no faunal analysis has been undertaken to assess the likelihood of this being the case. Taken together, the data indicate that the analyzed samples from Barcın Höyük may represent a minimum of 16 and a maximum of 19 individual animals.

Supplementary References

1. Kijas, J. W. *et al.* Genome-wide analysis of the world's sheep breeds reveals high levels of historic mixture and strong recent selection. *PLoS Biol.* **10**, e1001258 (2012).

2. Taylor, W. T. T. *et al.* Evidence for early dispersal of domestic sheep into Central Asia. *Nat. Hum. Behav.* (2021). doi:10.1038/s41562-021-01083-y

3. Skoglund, P. *et al.* Separating endogenous ancient DNA from modern day contamination in a Siberian Neandertal. *PNAS* **111**, 2229–2234 (2014).

4. Lv, F. H. *et al.* Mitogenomic meta-analysis identifies two phases of migration in the history of Eastern Eurasian sheep. *Mol. Biol. Evol.* **32**, 2515–2533 (2015).

5. Luo, Y.-Z. *et al.* Origin and genetic diversity of Mongolian and Chinese sheep using mitochondrial DNA D-loop sequences. *Acta Genet. Sin.* **32**, 1256–1265 (2005).

6. Singh, S., Kumar Jr, S., Kolte, A. P. & Kumar, S. Extensive variation and sub-structuring in lineage A mtDNA in Indian sheep: Genetic evidence for domestication of sheep in India. *PLoS One* **8**, e77858 (2013).

7. Gorkhali, N. A., Han, J. L. & Ma, Y. H. Mitochondrial DNA variation in indigenous sheep (Ovis aries) breeds of Nepal. *Trop. Agric. Res.* **26**, 632–641 (2015).

8. Demirci, S. *et al.* Mitochondrial DNA diversity of modern, ancient and wild sheep (Ovis gmelinii anatolica) from Turkey: New insights on the evolutionary history of sheep. *PLoS One* **8**, e81952 (2013).

9. Tapio, M. *et al.* Sheep mitochondrial DNA variation in European, Caucasian, and Central Asian areas. *Mol. Biol. Evol.* **23**, 1776–1783 (2006).

10. Ćinkulov, M. *et al.* Genetic differentiation between the Old and New types of Serbian Tsigai sheep. *Genet. Sel. Evol.* **40**, 321–331 (2008).

11. Pereira, F. *et al.* Genetic signatures of a Mediterranean influence in Iberian Peninsula sheep husbandry. *Mol. Biol. Evol.* **23**, 1420–1426 (2006).

12. Cohen, J. *Statistical power analysis for the social sciences*. (Lawrence Erlbaum Associates, 1988).

13. R Core Team. A language and environment for statistical computing. R Foundation for Statistical Computing, Vienna, Austria. https://www.r-project.org/. (2021).

14. Wickham, H. *ggplot2: Elegant graphics for data analysis*. (Springer, 2016).

15. RStudio Team. RStudio: Integrated Development Environment for R. RStudio, PBC, Boston, MA. http://www.rstudio.com/. (2020).

16. Alexander, D. H., Novembre, J. & Lange, K. Fast model-based estimation of ancestry in unrelated individuals. *Genome Res.* **19**, 1655–1664 (2009).

17. Behr, A. A., Liu, K. Z., Liu-Fang, G., Nakka, P. & Ramachandran, S. Pong: Fast analysis and visualization of latent clusters in population genetic data. *Bioinformatics* **32**, 2817–2823 (2016).

18. Meadows, J. R. S., Hiendleder, S. & Kijas, J. W. Haplogroup relationships between domestic and wild sheep resolved using a mitogenome panel. *Heredity.* **106**, 700–706 (2011).

19. Kumar, S., Stecher, G., Li, M., Knyaz, C. & Tamura, K. MEGA X: Molecular evolutionary genetics analysis across computing platforms. *Mol. Biol. Evol.* **35**, 1547–1549 (2018).

20. Hiendleder, S., Mainz, K., Plante, Y. & Lewalski, H. Analysis of mitochondrial DNA indicates that domestic sheep are derived from two different ancestral maternal sources: No evidence for contributions from Urial and Argali sheep. *J. Hered.* **89**, 113–120 (1998).

21. Uerpmann, H.-P. *The ancient distribution of ungulate mammals in the Middle East*. (Ludwig Reichert Verlag, 1987).

22. Zeder, M. A. Domestication and early agriculture in the Mediterranean Basin: Origins, diffusion, and impact. *PNAS* **105**, 11597–11604 (2008).

23. Zeder, M. A. Out of the fertile crescent: The dispersal of domestic livestock through Europe and Africa. in *Human Dispersal and Species Movement: From Prehistory to the Present* (eds. Boivin, N., Crassard, R. & Petraglia, M. D.) 261–303 (Cambridge University Press, 2017).

24. Zeder, M. A. The origins of agriculture in the Near East. *Curr. Anthropol.* **52**, S221–S235 (2011).

25. Rezaei, H. R. *et al.* Evolution and taxonomy of the wild species of the genus Ovis (Mammalia, Artiodactyla, Bovidae). *Mol. Phylogenet. Evol.* **54**, 315–326 (2010).

26. Nadler, C. F., Lay, D. M. & Hassinger, J. D. Cytogenetic analyses of wild sheep populations in northern Iran. *Cytogenetics* **10**, 137–152 (1971).

27. Valdez, R., Nadler, C. F. & Bunch, T. D. Evolution of wild sheep in Iran. *Evolution (N. Y).* **32**, 56–72 (1978).

28. Meadows, J. R. S., Cemal, I., Karaca, O., Gootwine, E. & Kijas, J. W. Five ovine mitochondrial lineages identified from sheep breeds of the near east. *Genetics* **175**, 1371–1379 (2007).

29. Geörg, C. *Paläopopulationsgenetik von Schwein und Schaf in Südosteuropa und Transkaukasien*. (VML Verlag Marie Leidorf, 2013).

30. Abazari, M. F. Investigation of mitochondrial hypervariable region I in an Iranian Neolithic livestock population: An exploration to understand the domestication origins. (2018).

31. Cai, D. *et al.* New ancient DNA data on the origins and spread of sheep and cattle in northern China around 4000 BP. *Asian Archaeol.* **2**, 51–57 (2018).

32. Feldman, M. *et al.* Late Pleistocene human genome suggests a local origin for the first farmers of central Anatolia. *Nat. Commun.* **10**, 1218 (2019).

33. Kılınç, G. M. *et al.* The demographic development of the first farmers in Anatolia. *Curr. Biol.* **26**, 2659–2666 (2016).

34. Keis, M. *et al.* Complete mitochondrial genomes and a novel spatial genetic method reveal cryptic phylogeographical structure and migration patterns among brown bears in north-western Eurasia. *J. Biogeogr.* **40**, 915–927 (2013).

35. Larson, G. & Burger, J. A population genetics view of animal domestication. *Trends Genet.* **29**, 197–205 (2013).

36. Paijmans, J. L. A., Gilbert, M. T. P. & Hofreiter, M. Mitogenomic analyses from ancient DNA. *Mol. Phylogenet. Evol.* **69**, 404–416 (2013).

37. Cai, D. W., Han, L., Zhang, X. L., Zhou, H. & Zhu, H. DNA analysis of archaeological sheep remains from China. *J. Archaeol. Sci.* **34**, 1347–1355 (2007).

38. Hiendleder, S., Lewalski, H., Wassmuth, R. & Janke, A. The complete mitochondrial DNA sequence of the domestic sheep (Ovis aries) and comparison with the other major ovine haplotype. *J. Mol. Evol.* **47**, 441–448 (1998).

39. Lancioni, H. *et al.* Phylogenetic relationships of three Italian Merino-derived sheep breeds evaluated through a complete mitogenome analysis. *PLoS One* **8**, e73712 (2013).

40. Hu, X. & Gao, L. The complete mitochondrial genome of domestic sheep, Ovis aries. *Mitochondrial DNA* **27**, 1425–1427 (2016).

41. Brahi, O. H. D., Xiang, H., Chen, X., Farougou, S. & Zhao, X. Mitogenome revealed multiple postdomestication genetic mixtures of West African sheep. *J. Anim. Breed. Genet.* **132**, 399–405 (2015).

42. Fan, H. *et al.* Complete mitochondrial genome sequences of Chinese indigenous sheep with different tail types and an analysis of phylogenetic evolution in domestic sheep. *Asian-Australalasian J. Anim. Sci.* **29**, 631–639 (2016).

43. Liu, J. B. *et al.* The complete mitochondrial genome sequence of the wild Huoba Tibetan sheep of the Qinghai-Tibetan Plateau in China. *Mitochondrial DNA Part A DNA Mapping, Seq. Anal.* **27**, 4689–4690 (2016).

44. Yang, C., Li, L., Zhong, T., Wang, L. & Zhang, H. Characterization of the complete mitochondrial genome sequence of Ujumuqin sheep (Ovis aries). *Mitochondrial DNA Part A DNA Mapping, Seq. Anal.* **28**, 315–316 (2017).

45. Niu, L. *et al.* Detecting signatures of selection within the Tibetan sheep mitochondrial genome. *Mitochondrial DNA Part A DNA Mapping, Seq. Anal.* **28**, 801–809 (2017).

46. Liu, R. *et al.* Analysis of mitochondrial DNA sequence and copy number variation across five high-altitude species and their low-altitude relatives. *Mitochondrial DNA Part B Resour.* **3**, 847–851 (2018).

47. Fu, D. *et al.* Characterization of the complete mitochondrial genome sequence of Jialuo sheep (Ovis aries). *Mitochondrial DNA Part B Resour.* **4**, 2116–2117 (2019).

48. Shepherd, L. D., Whitehead, P. & Whitehead, A. Genetic analysis identifies the missing parchment of New Zealand's founding document, the Treaty of Waitangi. *PLoS One* **14**, e0210528 (2019).

49. Deng, J. *et al.* Paternal origins and migratory episodes of domestic sheep. *Curr. Biol.* **30**, 4085-4095.e6 (2020).

50. Mustafa, S. I., Schwarzacher, T. & Heslop-Harrison, J. S. Complete mitogenomes from Kurdistani sheep: Abundant centromeric nuclear copies representing diverse ancestors. *Mitochondrial DNA Part A DNA Mapping, Seq. Anal.* **29**, 1180–1193 (2018).

51. Baird, D. *et al.* Juniper smoke, skulls and wolves' tails. The Epipalaeolithic of the Anatolian plateau in its South-west Asian context; insights from Pınarbaşı. *Levant* **45**, 175–209 (2013).

52. Baird, D. *et al.* Agricultural origins on the Anatolian plateau. *PNAS* **115**, E3077–E3086 (2018).

53. Baird, D. The late Epipaleolithic, Neolithic, and Chalcolithic of the Anatolian Plateau, 13,000 - 4000 BC. in *A Companion To The Archaeology Of The Ancient Near East* (ed. Potts, D. T.) 431–466 (Wiley-Blackwell, 2012).

54. Middleton, C. The beginning of herding and animal management: The early development of caprine herding on the Konya plain, central Anatolia. *Anatol. Stud.* **68**, 1–31 (2018).

55. Baird, D., Carruthers, D., Fairbairn, A. & Pearson, J. Ritual in the landscape: Evidence from Pınarbaşı in the seventh-millennium cal BC Konya Plain. *Antiquity* **85**, 380–394 (2011).

56. García-Suárez, A., Portillo, M. & Matthews, W. Early animal management strategies during the Neolithic of the Konya Plain, Central Anatolia: Integrating micromorphological and microfossil evidence. *Enviromental Archaeol.* **25**, 208–226 (2020).

57. French, D. H., Hillman, G. C., Payne, S. & Payne, R. J. Excavations at Canhasan III 1969-1970. in *Papers in Economic Prehistory* (ed. Higgs, E. S.) 181–194 (Cambridge University Press, 1972).

58. Fairbairn, A., Jacobsson, P., Baird, D., Jacobsen, G. & Stroud, E. Settlement change on the western Konya Plain: Refining Neolithic and Chalcolithic chronologies at Canhasan, Turkey. *Antiquity* **94**, 342–360 (2020).

59. Düring, B. S. *The prehistory of Asia Minor: From complex hunter-gatherers to early urban societies*. (Cambridge University Press, 2010).

60. Bıçakçı, E. *et al.* Les fouilles de Tepecik-Çiftlik et les activités du programme Melendiz préhistorique, campagne 2016. *Anatolia Antiq.* **25**, 71–94 (2017).

61. Çakan, Y. G. Neolitik Dönem'de yeni bir yapı tipi: Tepecik-Çiftlik'te "Fırınlı Yapılar". in *Arkeolojiyle Geçen Yarım Asır: Sevil Gülçur Armağanı* (eds. Çaylı, P., Demirtaş, I. & Eser, B.) 659–671 (Bilgin Kültür Sanat Yayınları, 2019).

62. Bıçakçı, E., Godon, M. & Çakan, Y. G. Tepecik-Çiftlik. in *The Neolithic in Turkey - Central Turkey* (eds. Özdoğan, M., Başgelen, N. & Kuniholm, P.) 89–134 (Archaeology and Art Publications, 2012).

63. Gündem, C. Y. MÖ 7. Binyılda Tepecik-Çiftlik Höyük'te hayvan ekonomisinin gelişimi ve evcil sığırın iç Anadolu Platosu'nda ortaya çıkışı. *Turkish Acad. Sci. J. Archaeol.* **25**, 28–45 (2019).

64. Godon, M. Cappadocia's first pottery production, Tepecik-Çiftlik Neolithic earliest levels. in *29. Araştırma Sonuçları Toplantısı* 317–340 (T.C. Kültür ve Turizm Bakanlığı Kültür Varlıkları ve Müzeler Genel Müdürlüğü, 2012).

65. Balcı, S. Tepecik-Çiftlik'te obsidiyen yongalama alanı. *Anadolu Prehistorya Araştırmaları Derg.* **2**, 148–159 (2016).

66. Balkan-Atlı, N. & Binder, D. Neolithic obsidian workshop at Kömürcü-Kaletepe (Cental Anatolia). in *The Neolithic in Turkey - Central Turkey* (eds. Özdoğan, M., Başgelen, N. & Kuniholm, P.) 71–88 (Archaeology and Art Publications, 2012).

67. Vinet, A. & Guilbeau, D. A first glimpse of the Late Neolithic and early Chalcolithic in Cappadocia through the Lithic industry of Tepecik-Çiftlik 2017 season. *Anatolia Antiq.* **XXVI**, 1–12 (2018).

68. Çevik, Ö. & Erdoğu, B. Absolute chronology of cultural continuity, change and break in Western Anatolia, between 6850-5480 calBC: The Ulucak case. *Mediterr. Archaeol. Archaeom.* **20**, 77–92 (2020).

69. Çakırlar, C. The evolution of animal husbandry in Neolithic central-west Anatolia: The zooarchaeological record from Ulucak Höyük (c. 7040–5660 cal. BC, Izmir, Turkey). *Anatol. Stud.* **62**, 1–33 (2012).

70. Guilbeau, D., Kayacan, N., Altınbilek-Algül, Ç., Erdoğu, B. & Çevik, Ö. A comparative study of the initial Neolithic chipped-stone assemblages of Ulucak and Uğurlu. *Anatol. Stud.* **69**, 1–20 (2019).

71. Milić, M. PXRF characterisation of obsidian from central Anatolia, the Aegean and central Europe. *J. Archaeol. Sci.* **41**, 285–296 (2014).

72. Çevik, Ö. & Abay, E. Neolithisation in Aegean Turkey: Towards a more realistic reading. in *Anatolian Metal VII: Anatolien und Seine Nachbarn vor 10.000 Jahren/Anatolia and Neighbours 10,000 Years Ago* (ed. Yalçın, Ü.) 199–209 (Dt. Bergbau Museum, 2016).

73. Çevik, Ö. Changing ideologies in community-Making through the Neolithic period at Ulucak. in *Concluding the Neolithic: The Near East in the Second Half of the Seventh Millennium BCE* (ed. Marciniak, A.) 219–239 (Lockwood Press, 2019).

74. Zeder, M. A. & Pilaar, S. E. Assessing the reliability of criteria used to identify mandibles and mandibular teeth in sheep, Ovis, and goats, Capra. *J. Archaeol. Sci.* **37**, 225–242 (2010).

75. Zeder, M. A. & Lapham, H. A. Assessing the reliability of criteria used to identify postcranial bones in sheep, Ovis, and goats, Capra. *J. Archaeol. Sci.* **37**, 2887–2905 (2010).

76. Grant, A. The use of tooth wear as a guide to the age of domestic ungulates. in *Ageing and sexing animal bones from archaeological sites* (eds. Wilson, B., Grigson, C. & Payne, S.) 91–108 (BAR Publishing, 1982).

77. Silver, I. A. The ageing of domestic animals. in *Science in Archaeology: A Survey of Progress and Research* (eds. Brothwell, D. & Higgs, E.) 283–302 (Thames and Hudson, 1969).

78. von den Driesch, A. *A guide to the measurement of animal bones from archaeological sites*. (Peabody Museum Press, 1976).

79. Gerritsen, F. & Özbal, R. Barcın Höyük, a seventh millennium settlement in the Eastern Marmara region of Turkey. *Doc. Praehist.* **46**, 58–67 (2019).

80. Özbal, R. & Gerritsen, F. Barcın Höyük in interregional perspective: A first assessment. in *Concluding the Neolithic: The Near East in the Second Half of the Seventh Millennium BCE* (ed. Marciniak, A.) 287–306 (Lockwood Press, 2019).

81. Gerritsen, F., Özbal, R. & Thissen, L. The earliest Neolithic levels at Barcın Höyük, Northwestern Turkey. *Anatolica* **39**, 53–92 (2013).

82. Özbal, H. *et al.* Neolitik Batı Anadolu ve Marmara yerleşimleri çanak çömleklerinde organik kalıntı analizleri. in *28. Arkeometri Sonuçları Toplantısı* 105–114 (T.C. Kültür ve Turizm Bakanlığı Kültür Varlıkları ve Müzeler Genel Müdürlüğü, 2013).

83. Özbal, R. & Gerritsen, F. Farmer-forager interactions in the Neolithisation of Northwest Anatolia: Reassessing the evidence. in *Proceedings of the international workshop held at the 10ICAANE Conference in Vienna, April 2016* (eds. Brami, M. & Horejs, B.) 181–210 (Austrian Academy of Sciences Press, 2019).

84. de Groot, B., Thissen, L. & Özbal, R. Clay preparation and function of the first ceramics in north-west Anatolia: A case study from Neolithic Barcın Höyük. *J. Archaeol. Sci. Reports* **16**, 542–552 (2017).

85. Thissen, L., Özbal, H., Türkekul-Bıyık, A., Gerritsen, F. & Özbal, R. The land of milk? Approaching, dietary preferences of Late Neolithic communities in NW Anatolia. *Leiden J. Pottery Stud.* **26**, 157–172 (2010).

86. Balcı, H., Cappers, R. T. J., Gerritsen, F. & Özbal, R. Barcın Höyük'te bitki seçimi: 2013-2015 yılı arkeobotanik sonuçlarının değerlendirilmesi. in *34. Arkeometri Sonuçları Toplantısı* 333–352 (T.C. Kültür ve Turizm Bakanlığı Kültür Varlıkları ve Müzeler Genel Müdürlüğü, 2019).

87. Würtenberger, D. A. Archäozoologische Analysen am Fundmaterial des Barcın Höyüks im Vergleich mit ausgewählten Fundstellen des 7. und 6. Jt. v. Chr. in Nordwest- und Westanatolien. (University of Vienna, 2012).
