# Supplementary material for: Archaeogenetic analysis of Neolithic sheep from Anatolia suggests a complex demographic history since domestication
Source: Commun Biol. 2021 Nov 12;4:1279. doi: 10.1038/s42003-021-02794-8 (PMC8589978; doi:10.1038/s42003-021-02794-8)
Supplement: Supplementary file 3 — Description of Additional Supplementary Files [file 42003_2021_2794_MOESM3_ESM.pdf]

Description of Additional Supplementary Files

File name: Supplementary Data

Supplementary Data 1: Genetic and archaeological information on sheep used in nuclear and mitochondrial DNA analyses. The "Date" column shows the age of samples estimated either based on archaeological context, direct C14 dates for 5 individuals, which are shown in bold. The direct date information includes (1) sigma calibrated age estimates (cal BCE), (2) conventional radiocarbon ages before present (BP) marked with δ, and (3) AMS laboratory codes in parentheses. "Number of times sequenced" indicates how many 144-bp mtDNA fragments were produced by Sanger sequencing. "HPG" shows haplogroup assignment based on the 144-bp mtDNA fragment. The four samples marked with asterisks (*) in the third column are those where we could generate sufficient nuclear genome data (see Supplementary Table 2). TEP3_depo, TEP58, TEP62, TEP83 and ULU31 were directly dated in this study. The tag 'depo' indicates samples were repository specimens. Haplogroup assignments were revised based on mitogenome data generated by high-throughput for samples TEP03 and ULU31; the revised assignments are shown in parenthesis.

Supplementary Data 2: Sequencing and mapping statistics for ancient sheep libraries sequenced on the Illumina HiSeq platform. The samples include material from 3 sites: Ulucak Höyük (uhs), Tepecik-Çiftlik Höyük (tps) and Barcın Höyük (bhs). The reported sheep proportions were calculated based on the proportion of all reads mapping to the sheep genome, before filtering for mismatches or removing duplicates. Genome coverage was calculated using reads remaining after filtering and duplicate removal. Sequencing IDs marked by asterisks (*) (*n*=5) refer to SNP-capture libraries. All of the five SNP-capture libraries were used for mtDNA HPG analyses whereas all except ULU26 were used in population genomic analyses using nuclear data. ULU26 was excluded from the downstream analyses due to low SNP count.

Supplementary Data 3: The 20,000 SNPs used in the SNP capture protocol. The table lists chromosomal locations (in Oar_v3.1 coordinates), as well as reference and alternative alleles of each SNP. The list includes 8850 transversions and 11150 transitions (*i.e.* G/A and C/T alleles). 18,575 are autosomal, 1,472 are on chrX, and 3 are mitochondrial. The list is a subset of the Illumina OvineSNP50 Beadchip variant set.

Supplementary Data 4: *D*-statistics results using the whole data. OAM8 is an Argali sheep individual. representing the outgroup while ANG represents goat. **A)** *D*-statistics results of the form *D(Goat, Argali;* $Modern_1$*,* $Modern_2$*)*. **B)** *D*-statistics results of the form *D(Outgroup,* ANS_1*;* ANS_2*, Modern)*. **C)** *D*-statistics results of the form *D(Outgroup, Modern;* ANS_1*,* ANS_2*)*. **D)** *D*-statistics results of the form *D(Outgroup,* ANS_1*;* ANS_2*,* ANS_3*)*. **E)** *D*-statistics results of the form *D(Outgroup, ANS; European, Non-European)*. **F)** *D*-statistics results of the form *D(Outgroup, OBI;* $Modern_1$*,* $Modern_2$*)*. **G)** *D*-statistics results of the form *D(Outgroup, Modern; OBI, ANS)*. **H)** *D*-statistics results of the form *D(Outgroup,* $Modern_1$*;* $Modern_2$*, ANS)*. **I)** *D*-statistics results of the form *D(Outgroup,* $Modern_1$*;* $Modern_2$*, OBI)*. **J)** *D*-statistics results of the form *D(Outgroup, OBI; ANS, Modern)*. **K)** *D*-statistics results of the form *D(Outgroup, ANS; OBI, Modern)*.

Supplementary Data 5: Eigenvalues of first 10 principal components of the PCA calculated using modern sheep breeds, ANS and OBS.

Supplementary Data 6: Outgroup f_3-statistics results using the whole data. OAM8 is an Argali sheep individual representing the outgroup. **A)** Outgroup f_3-statistics results of the form f_3*(Outgroup; Modern, ANS)*. **B)** Outgroup f_3-statistics results of the form f_3*(Outgroup; Modern,* $ANS_1/ANS_2/ANS_3$*)*. **C)** Outgroup f_3-statistics results of the form f_3*(Outgroup; Modern, OBI)*.

Supplementary Data 7: *D*-statistics results using only transversions. OAM8 is an Argali sheep individual. representing the outgroup and ANG represents goat. **A)** *D*-statistics results of the form *D(Goat, Argali;* $Modern_1$*,* $Modern_2$*)*. **B)** *D*-statistics results of the form *D(Outgroup,* ANS_1*;* ANS_2*, Modern)*. **C)** *D*-statistics results of the form *D(Outgroup, Modern;* ANS_1*,* ANS_2*)*. **D)** *D*-statistics results of the form *D(Outgroup,* ANS_1*;* ANS_2*,* ANS_3*)*. **E)** *D*-statistics results of the form *D(Outgroup, ANS; European, Non-European)*. **F)** *D*-statistics results of the form *D(Outgroup,* $Modern_1$*;* $Modern_2$*, ANS)*.

Supplementary Data 8: Outgroup f_3-statistics results of the form f_3*(Outgroup; Modern, ANS)* using only transversions. OAM8 is an Argali sheep individual representing the outgroup.

Supplementary Data 9: NJ tree constructed by 310 modern samples and 5 ancient samples in newick format.

Supplementary Data 10: Haplogroups of 315 mitogenomes, assigned by NJ and 144 bp analyses.
