# Supplementary material for: Archaeogenetic analysis of Neolithic sheep from Anatolia suggests a complex demographic history since domestication
Source: Commun Biol. 2021 Nov 12;4:1279. doi: 10.1038/s42003-021-02794-8 (PMC8589978; doi:10.1038/s42003-021-02794-8)
Supplement: Supplementary file 5 — Reporting Summary [file 42003_2021_2794_MOESM5_ESM.pdf]

nature portfolio

Corresponding author(s): Mehmet Somel, Füsun Özer

Last updated by author(s): Aug 27, 2021

Reporting Summary

Nature Portfolio wishes to improve the reproducibility of the work that we publish. This form provides structure for consistency and transparency in reporting. For further information on Nature Portfolio policies, see our Editorial Policies and the Editorial Policy Checklist.

Statistics

For all statistical analyses, confirm that the following items are present in the figure legend, table legend, main text, or Methods section.

n/a	Confirmed	
☐	☒	The exact sample size (n) for each experimental group/condition, given as a discrete number and unit of measurement
☐	☒	A statement on whether measurements were taken from distinct samples or whether the same sample was measured repeatedly
☐	☒	The statistical test(s) used AND whether they are one- or two-sided<br>*Only common tests should be described solely by name; describe more complex techniques in the Methods section.*
☒	☐	A description of all covariates tested
☐	☒	A description of any assumptions or corrections, such as tests of normality and adjustment for multiple comparisons
☐	☒	A full description of the statistical parameters including central tendency (e.g. means) or other basic estimates (e.g. regression coefficient) AND variation (e.g. standard deviation) or associated estimates of uncertainty (e.g. confidence intervals)
☐	☒	For null hypothesis testing, the test statistic (e.g. F, t, r) with confidence intervals, effect sizes, degrees of freedom and P value noted<br>*Give P values as exact values whenever suitable.*
☒	☐	For Bayesian analysis, information on the choice of priors and Markov chain Monte Carlo settings
☒	☐	For hierarchical and complex designs, identification of the appropriate level for tests and full reporting of outcomes
☐	☒	Estimates of effect sizes (e.g. Cohen's d, Pearson's r), indicating how they were calculated

Our web collection on statistics for biologists contains articles on many of the points above.

Software and code

Policy information about availability of computer code

Data collection	No software was used for data collection.
Data analysis	The software used in this study: AdapterRemoval (v.2.3.1), BWA (v. 0.7.15), SAMtools (v. 1.9), FilterUniqueSAMCons.py, bamUtil (v.1.0.14), UCSC Genome Browser liftover tool, PMDtools (v.0.60), R (v.3.5), Rstudio (v. 3.5), PLINK (v. 1.9), EIGENSOFT (v. 7.2.0), AdmixTools (v. 5.1), ADMIXTURE (v.1.3), Pong (v.1.4.9), BCFtools (v.1.9), Python 3, MEGA-X.

For manuscripts utilizing custom algorithms or software that are central to the research but not yet described in published literature, software must be made available to editors and reviewers. We strongly encourage code deposition in a community repository (e.g. GitHub). See the Nature Portfolio guidelines for submitting code & software for further information.

Data

Policy information about availability of data

All manuscripts must include a data availability statement. This statement should provide the following information, where applicable:

- Accession codes, unique identifiers, or web links for publicly available datasets
- A description of any restrictions on data availability
- For clinical datasets or third party data, please ensure that the statement adheres to our policy

All .fastq files were submitted to the European Nucleotide Archive (ENA) with reference number PRJEB36540.
All ancient mitochondrial DNA sequences produced in this study have been deposited in the NCBI Genbank database (accession number MT321187- MT321260).

Field-specific reporting

Please select the one below that is the best fit for your research. If you are not sure, read the appropriate sections before making your selection.

☐ Life sciences ☐ Behavioural & social sciences ☒ Ecological, evolutionary & environmental sciences

For a reference copy of the document with all sections, see nature.com/documents/nr-reporting-summary-flat.pdf

Ecological, evolutionary & environmental sciences study design

All studies must disclose on these points even when the disclosure is negative.

Study description	This study is about understanding the demographic history of Anatolian Neolithic sheep by combining analyses of nuclear polymorphism data and mitochondrial DNA data. We generated genome-wide ancient DNA data from four samples. These genotypes were compared among themselves and to published 50K SNPchip genotype data from modern breeds from Anatolia, Europe and Southwest Asia (Kijas et al., 2012) and central Asian Neolithic/Bronze Age sheep from Kyrgyzstan (Taylor et al., 2021). Genome-wide analysis show Anatolian Neolithic sheep are genetically closest to modern European breeds, meanwhile and central Asian Neolithic/Bronze Age sheep is genetically closer to modern Asian breeds. Our results suggest that European and Anatolian domestic sheep gene pools have been strongly remolded since the Neolithic.
Research sample	Ancient DNA samples extracted from Anatolian early Holocene sheep bone material.
Sampling strategy	We used samples collected earlier by zooarchaeology teams from the field during ongoing excavations or from the storage rooms of the arcaheological sites where the previous years' samples were stored. Determining the sample size a priori was not required for this study. Due to the nature of ancient DNA not all samples provide enough DNA. Therefore, all samples that yielded sufficient DNA were included in the study.
Data collection	Genome sequencing was carried out in collaboration with Stockholm University, on Illumina Hiseq 4000 and Hiseq X Platforms. Mitochondrial DNA data collection was performed on the ABI 3100 Genetic Analyzer at the core facilities of Refgen Inc (Ankara, Turkey) and Macrogen (Amsterdam, the Netherlands).
Timing and spatial scale	We used early Holocene (mainly Neolithic period) sheep samples from Central and West Anatolia. These samples were chosen because the central Anatolian samples lie within the vicinity of the primary domestication centers, west Anatolian samples were chosen to understand spread of sheep to the west of Anatolian and to Europe whereas central Asian Neolithic/Bronze Age sheep were chosen to understand relationship between ancient Asian and modern breeds. Five individuals Anatolian bones were C14 dated to c.7000-6000 BCE and three previously published central Asian bones were dated as 7,012-4,260 BCE.
Data exclusions	None of the data is excluded.
Reproducibility	We could replicate our experimental results at every stage from wet laboratory to computational analysis. Specifically, in mtDNA analyses we used large sample sizes and only fragments that could be sequenced twice were included. In demographic analyses using genomes, we used both multiple individuals representing a population and also information from across the genome to conclude about statistically significant patterns. In analyses including genomic polymorphism data, we further tested for reproducibility of demographic patterns using only molecules bearing post-mortem damage signatures.
Randomization	This study did not involve an experimental design or a design that could lead to batch effects; therefore randomization was not applied.
Blinding	This study did not involve an experimental design or a design that could lead to batch effects; therefore blinding was not applied.

Did the study involve field work? ☐ Yes ☒ No

Reporting for specific materials, systems and methods

We require information from authors about some types of materials, experimental systems and methods used in many studies. Here, indicate whether each material, system or method listed is relevant to your study. If you are not sure if a list item applies to your research, read the appropriate section before selecting a response.

Materials & experimental systems

n/a	Involved in the study	
☒	☐	Antibodies
☒	☐	Eukaryotic cell lines
☐	☒	Palaeontology and archaeology
☒	☐	Animals and other organisms
☒	☐	Human research participants
☒	☐	Clinical data
☒	☐	Dual use research of concern

Methods

n/a	Involved in the study	
☒	☐	ChIP-seq
☒	☐	Flow cytometry
☒	☐	MRI-based neuroimaging

Palaeontology and Archaeology

Specimen provenance	The specimens used in this study had been previously unearthed from the archaeological excavation sites by expert zooarchaeologists and provided to the researchers by each excavation director's written permission.
Specimen deposition	The specimens have been deposited in the ancient DNA laboratory at the Middle East Technical University.
Dating methods	New C14 dates for six specimen were obtained in this study. 2-5 grams of specimen were cut and surface cleaned prior to sending to the analysing laboratories. Five samples were AMS C14 dated at the TÜBİTAK-MAM (Gebze, Turkey) and one sample at Beta Analytic Inc. (London, UK). Radiocarbon ages were calibrated using the INTCAL13 database. The quality assurance reports were provided by the above laboratories.

☒ Tick this box to confirm that the raw and calibrated dates are available in the paper or in Supplementary Information.

Ethics oversight	All permits for archaeological samples processed in this study were obtained from relevant museums affiliated to Republic of Turkey Ministry of Culture and Tourism.

Note that full information on the approval of the study protocol must also be provided in the manuscript.
